# The Tandem Ring Opening/Ring Closing Metathesis Route to Oxaspirocycles: An Approach to Phelligridin G

**DOI:** 10.3390/molecules18022438

**Published:** 2013-02-21

**Authors:** Harold D. Cooper, Dennis L. Wright

**Affiliations:** 1 Department of Pharmaceutical Sciences, University of Connecticut, Storrs, CT 06269, USA; E-Mail: Harold.cooper@uconn.edu; 2 Department of Chemistry, University of Connecticut, Storrs, CT 06269, USA

**Keywords:** phelligridin G, Diels-Alder, spiroannulations, metathesis, 2-phenylfurans, natural products

## Abstract

Phelligridin G is an unusual natural product that contains an embedded spiro-fused furanone core. We have investigated two furan-based synthetic approaches towards the spirocyclic core structure of this natural product from readily available 2-phenylfurans. Although initial studies involving an oxidative cyclization were unsuccessful, we were ultimately able to access this key system through a sequential intermolecular furan Diels-Alder reaction followed by a metathesis-based reorganization. A related approach led to an expanded C ring to form spiro-fused pyran spirocycles.

## 1. Introduction

**Figure 1:**
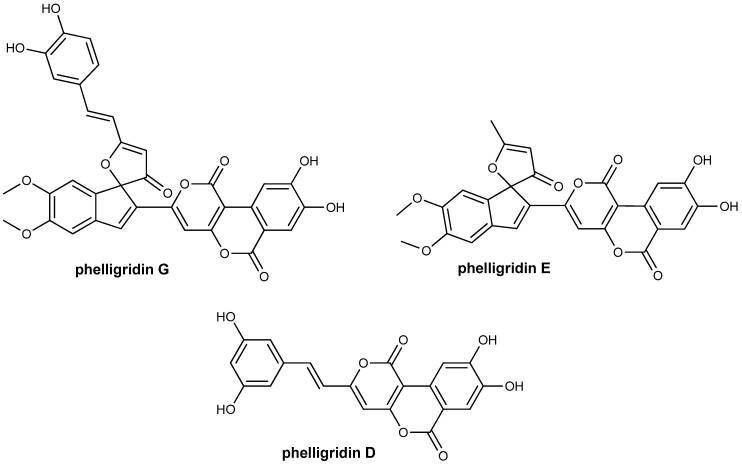
Structural Derivatives of *Phellinus igniarius*.

There are a variety of therapeutics in use today that are derived from natural products isolated from various plant, marine or microbial sources. There is increasing evidence from animal studies that regular administration of powdered medicinal mushrooms can produce anti-cancer effects, demonstrating both high antitumor activity and restriction of tumor metastasis [1]. One such family of medicinal fungi has been derived from the species *Phellinus igniarius*, a member of the Polyporaceae family. Phelligridins D, E and G (Figure 1) are interesting natural products isolated from the fruiting body of *P. igniarius*. The fruiting body of *P. igniarius* has been long used in its role as a Traditional Chinese Medicine for the treatment of fester, bellyache and bloody gonorrhea [2]. Phelligridin G displays significant antioxidant activity, inhibiting rat liver microsomal lipid peroxidation and also shows moderate selective cytotoxic activity against a human ovarian (A2780) and colon cancer cell line (HCT-8) [3]. Based on the potential of phelligridin G as an anti-cancer agent, along with its unique and synthetically challenging spirofused-furanone, we set out to develop a rapid and efficient synthetic route to the core domain of the natural product.

**Scheme 1:**
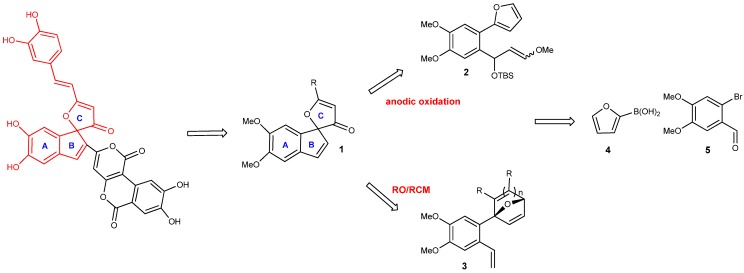
Retrosynthetic analysis of Phelligridin G.

Our retrosynthetic analysis targeted the core domain **1**, comprised of the A, B and C rings characterized by the challenging 2-oxaspiro-[4.4]-octane unit (Scheme 1). We have been interested for some time in developing furan-based methodologies for the synthesis of polycyclic systems and felt that phelligridin G offered an excellent target to evaluate two strategies we have pursued. In one path, the presence of the spiro-fused 3-furanone makes it an attractive target for an electrochemical annulation strategy through oxidative cyclization of **2**. We have worked extensively with furan-terminating electrochemical oxidative cyclizations and this method has been successful in the formation of 5-, 6-, and 7-membered carbocycles [4,5,6,7]. In a parallel approach to the anodic oxidation, a sequential furan Diels-Alder reaction to give **3**, followed by a ring opening metathesis/ring closing metathesis (ROM/RCM) process could also deliver the target intermediate, a strategy we have recently employed in an approach to cyclopamine [8]. Exploration of both strategies would rely on the same two commercially available building blocks **4** and **5**.

## 2. Results and Discussion

### 2.1. Electrochemical Studies

**Scheme 2:**
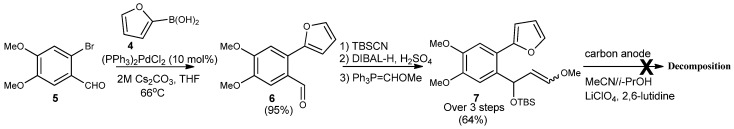
Attempted oxidative cyclization to the spirocyclic core.

The commercially available starting materials 2-furanboronic acid (**4**) and 3,4-dimethoxybromo-benzaldehyde (**5**) were joined through standard Suzuki cross-coupling with bis(triphenylphosphine)-palladium(II) dichloride to produce the 2-phenylfuran derivative **6** in good yield (Scheme 2). The aldehyde was homologated with *t*-butyldimethylsilyl cyanide, followed by reduction of the nitrile group in the presence of DIBAL-H to give the protected α-hydroxyaldehyde after hydrolysis with dilute sulfuric acid. The α,β-unsaturated aldehyde underwent smooth Wittig olefination to deliver enol ether intermediate **7**. Unfortunately, and despite earlier success with similar systems [9], all attempts to produce the spiro-fused product through anodic oxidation failed, producing only complex mixtures of products. Further oxidative studies were attempted using the traditional chemical oxidants cerium(IV)-ammonium nitrate (CAN) [10] and tris(4-bromophenyl)aminium hexachloroantimonate (BAHA) [11] which are known to oxidize electron-rich olefins, but these also proved to be ineffective. We believe that the presence of additional groups with low oxidation potentials such as the dimethoxyphenyl ring and a 2-aryl furan leads to significant over oxidation and ultimate destruction of the starting material. The failure to detect any appreciable cyclized material prompted us to explore the alternative cycloaddition/metathesis approach to this class of compounds.

### 2.2. Diels-Alder/Metathesis Studies

**Scheme 3:**
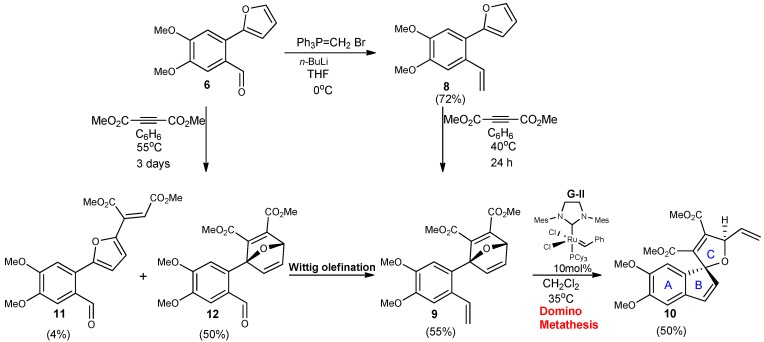
Synthesis of ABC spiroannulated core via Domino ROM/RCM metathesis.

The value of oxabicyclic building blocks for the preparation of five-membered heterocycles has been appreciated for some time [12,13,14]. Our synthesis of the spirocyclic core of phelligridin G would be achieved in four steps starting with the previously described furyl-benzaldehyde derivative **6** (Scheme 3). Olefination of the α,β-unsaturated carbonyl with methyl triphenylphosphonium bromide in the presence of *n*-butyl lithium yielded styrylfuran **8**. In order to obtain a suitable precursor for the metathesis-driven bond reorganization, thermal cycloaddition to the furan via standard Diels-Alder reaction was envisioned. Usually furan is a highly reactive diene, but in this system this reactivity became highly attenuated due to the presence of the aryl ring in the C2 position. It has been previously observed that 2-phenylfurans are poor dienes in Diels-Alder cycloadditions as a direct result of their high π–conjugation, which is destroyed upon formation of the cycloadduct, thus promoting rapid retro-Diels-Alder reaction [15]. We explored a series of structurally diverse dienophiles based upon accessibility and reactivity rates to form the cycloaddition products.

We found that the sp^2^-type dienophiles such as *n*-methylmaleimide, cyanovinyl acetate, dimethyl fumarate, and fumaryl chloride only returned unchanged starting materials after days of continuous heating. Using more reactive alkynes seemed promising as we had some success with treating substituted furan scaffolds with acetylenic dienophiles in previous studies [8]. However, with this 2-phenylfuran no oxabicyclic ether formation was observed at moderate temperatures with monofunctionalized alkynes such as tosyl acetylene or methyl propriolate. At higher temperatures, cycloaddition does occur, but this is followed by a spontaneous ring-opening/aromatization process to produce an undesired phenol. Pleasingly, use of a more deactivated alkyne, dimethyl acetylenedicarboxylate (DMAD), under mild thermal conditions (rt→40 °C) led to the desired adduct **9** in reasonable yield. We also examined the potential of aldehyde **6** to participate in the [4+2] cycloaddition. As expected, condensation of aldehyde **6** with DMAD required higher temperatures (55 °C) as well as longer reaction time, owing to the presence of the electron-withdrawing group which deactivates the furan ring system. Ultimately, this reaction did produce the adduct **12** in ~50% isolated yield, which could be taken on to **9** by olefination. Furthermore, small amounts of Michael product **11** occurred as a byproduct. With the key oxabridged intermediate in hand, we were able to attempt the key metathesis reaction. The oxabicyclo adduct **9** undergoes a ring opening/ring closing (ROM/RCM) domino type metathesis using Grubbs 2^nd^ generation catalyst to form spiro compound **10** as a single diastereomer (as observed by ^1^H and ^13^C-NMR) that contains the core structure of phelligridin G. The release of ring-strain in the bridged bicyclic compound is an important driving force in these types of reactions and have been increasingly used to generate complex natural products [8,16,17,18,19].

**Scheme 4:**
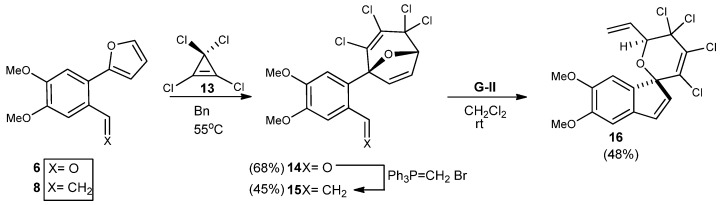
Expansion of the C-ring of the phelligridin G core to form the spiro-fused pyran moiety.

In our desire to expand this utility of this methodology, we looked at the highly reactive perhalogenated cyclopropenes as cycloaddition partners (Scheme 4). With the assistance of these compounds, we have been able to develop building blocks leading to the synthesis of several natural products [20,21]. It was felt that the cycloaddition of perhalocyclopropenes would be more efficient as the initial cycloadduct spontaneously rearranges to the ring-expanded system thus preventing the retro-Diels-Alder reaction from taking place. Oxabicyclo[3.2.1]octane **14** was easily prepared in a one-pot sequence that involved heating **6** and tetrachlorocyclopropene (TCCP) **13** at 55 °C for 4 days, followed by olefination to give **15** without benefit of further purification. Compound **8** under similar reaction conditions was much less reactive as the alkene seemed to hinder the cycloadduct formation with this particular dienophile.

The strained ether-bridged seven-membered ring likely undergoes a ring opening of the endocyclic double bond followed by closure with the terminal olefin to form the B ring of the spiro-fused product. For oxabicyclo[3.2.1]octane **15**, the **GII** catalyst was also optimal but heating of the mixture was unnecessary in this system and produce the highly functionalized spiro-pyran **16** in moderate overall yield.

## 3. Experimental

### General

All reactions were carried out under an inert argon atmosphere with dry solvents under anhydrous conditions, unless otherwise noted. Commercial grade reagents and solvents were purchased and used without further purification except as indicated below. Hexanes, tetrahydrofuran (THF), diethyl ether (Et_2_O), and dichloromethane (CH_2_Cl_2_) were used directly as obtained from a Baker Cycle-tainer™ system. Yields refer to chromatographically and spectroscopically (^1^H-NMR) homogenous materials, unless otherwise stated. Reactions were monitored by thin layer chromatography (TLC) carried out on Whatman silica gel 60 Å precoated plates using UV light as the visualizing agent and an acidic mixture of DNP or basic aqueous potassium permanganate (KMnO_4_) and heat as developing agents. Flash chromatography was performed using Baker silica gel (60 Å particle size). NMR spectra were recorded on Bruker-500 and 400 instruments and calibrated using the residual undeuterated solvent signal as an internal reference (CHCl_3_ at δ 7.26 ppm for ^1^H-NMR, δ 77.1 ppm for ^13^C-NMR). The following abbreviations were used to explain the multiplicities: s = singlet, d = doublet, t = triplet, q = quartet, m = multiplet, b = broad. IR Spectra were recorded on Shimadzu FT-IR 8400 spectrometer. Melting points (m.p.) were uncorrected and measured with a Mel-Temp digital melting point apparatus. High resolution mass spectra (HRMS) were obtained from the University of Connecticut Mass Spectral Facility by using Direct Analysis in Real Time using an AccuTOF (JEOL) mass spectrometer.

*2-Furan-2-yl-4,5-dimethoxybenzaldehyde* (**6**). Commercially available 3,4-dimethoxybromo-benzaldehyde **5** (0.5 g, 2.04 mmol) was added to an argon purged 25 mL flask. Anhydrous THF (10 mL) was added, followed by 2-furanboronic acid (2 equiv.), and PdCl_2_(PPh_3_)_2_ (0.1 mol equiv.). Once all the reagents were in solution, 2M aqueous solution of Cs_2_CO_3_ (3 mol equiv.) was added to the flask, which was immediately placed in a preheated oil bath and refluxed with vigorous stirring for 12–24 h. Once the reaction was deemed complete, it was cooled to room temperature, quenched with H_2_O, and diluted with Et_2_O. The aqueous layer was extracted with Et_2_O (3 × 5 mL) and the combined organic layers were washed with brine, dried over Na_2_SO_4_, and concentrated to give a crude yellow solid. The crude product was purified by column chromatography on silica gel (6:1 hexane/ethyl acetate) to yield **6** (95%) as a solid. R*_f_* = 0.38; m.p. = 102 °C; IR (KBr, cm^−1^): *υ*: 730, 909, 1280, 2255, 3012 ^1^H-NMR (400 MHz, CDCl_3_): δ = 3.96–4.04 (m, 6H), 6.58 (s, 2H), 7.13 (s, 1H), 7.54 (s, 1H), 7.62 (s, 1H), 10.27 (s, 1H). ^13^C-NMR (126 MHz, CDCl_3_) δ = 190.58, 154.46, 153.48, 150.43, 149.04, 143.47, 128.85, 126.67, 115.40, 111.76, 110.38, 108.83, 77.44, 77.12, 76.80, 65.76, 56.00; HRMS (DART) calcd. for C_13_H_13_O_4_ [M+H]^+^: 233.0814; found 233.0823. 

*(tert-Butyldimethylsilanyloxy)-2-furan-2’-yl-‘4,5’-dimethoxyphenyl)acetonitrile.* To a flame dried flask was added *t*-butyldimethylsilyl cyanide (0.141 g, 1.0 mmol), potassium cyanide (0.003 g, 0.05 mmol), and 18-crown-6 (0.053 g, 0.4 mmol) under an argon atmosphere. A solution of aldehyde **6** (0.116 g, 0.500 mmol) in dry methylene chloride (5 mL) was added dropwise over 30 min to the homogenous mixture. The reaction was stirred vigorously for 24 h and monitored by TLC. Upon reaction completion the solvent is removed *in vacuo* and the crude oily residue was purified by flash chromatography using 15% EtOAc in hexanes to obtain a pure yellow oil (0.177g, 95% yield). R*_f_* = 0.67; IR (KBr, cm^−1^) *υ*: 654, 735, 908, 2254, 2962, 3012 ^1^H-NMR (500 MHz, CDCl_3_): δ = 0.02–0.12 (d, *J* = 50 Hz, 6H), 0.89 (s, 9H), 3.93–3.95 (d, *J* = 10 Hz, 6H), 5.89 (s, 1H), 6.53 (s, 2H), 7.02 (s, 1H), 7.30 (s,1H), 7.55 (s, 1H). ^13^C-NMR (126 MHz, CDCl_3_) δ = 151.8, 149.5, 147.2, 126.6, 121.8, 119.7, 111.8, 110.9, 110.1, 108.9, 61.2, 56.2, 25.7, 18.3, −5.1; HRMS (DART) calcd. for C_20_H_28_ NO_4_Si [M+H]^+^: 374.1788; found 374.1776. 

*(tert-Butyldimethylsilanyloxy)-2-furan-2’-yl-‘4,5’-dimethoxyphenyl)acetaldehyde.* Silyl cyanide (0.455 g, 1.22 mmol) was dissolved in dry toluene (4 mL) and cooled to −78 °C. DIBAL (0.868 g, 6.1 mmol) was added dropwise to the flask and the mixture stirred 2 h at this temp. Once the imine is formed, the reaction was quenched with methanol (2.1 mL) and warmed up to 0 °C. After 2 h, 1N H_2_SO_4_ (2 mL) was added dropwise to the mixture while stirring for 1 h at this temperature. Diethyl ether was added to dilute the reaction and the aqueous layer was separated. The aqueous layer was extracted with ether (3 × 3 mL) and the ethereal layer collected, washed with brine, dried over Na_2_SO_4_, and the solvent removed *in vacuo*. The crude oil was purified by flash chromatography using a 10% EtOAc in hexanes solution to obtain a pure oil (0.200 g, 44% yield). R*_f_* = 0.49; IR (KBr, cm^−1^) *υ*: 732, 913, 1735, 2934, 2959, 3012 ^1^H-NMR (500 MHz, CDCl_3_): δ= −0.09−0.02 (d, *J* = −35 Hz, 6H), 0.87 (s, 9H), 3.91–3.92 (d, *J* = 5 Hz, 6H), 5.50 (s, 1H), 6.49–6.61 (dd, *J* = 10, 5 Hz, 2H), 7.07–7.09 (d, *J* = 10 Hz, 2H), 7.51 (s, 1H). ^13^C-NMR (126 MHz, CDCl_3_) δ = 198.9, 152.3, 149.3, 148.9, 142.4, 127.2, 122.8, 111.5, 111.2, 110.5, 108.9, 76.2, 56.0, 25.7, 25.7, 18.3, −4.9; HRMS (DART) calcd. for C_20_H_29_O_5_Si [M+H]^+^: 377.1784; found 377.1760. 

*tert-Butyl-[1-(2-furan-2’yl-4’,5’-dimethoxyphenyl)-3-methoxyallyloxy]dimethylsilane* (**7**). To a stirred solution of dry THF (3 mL) was added methoxymethyltriphenylphosphonium bromide (0.36 g, 0.105 mmol) under argon. The homogenous mixture was cooled to 0 °C followed by addition of NaO*t-*Bu base (2 eq.) in one portion. The red-orange suspension was stirred about 30 min as the color persisted under the above temp. A 0.20 mM solution of 2-furyl benzaldehyde (0.802 g, 3.45 mmol) in THF was slowly added, and the reaction was monitored by TLC at 0 °C. The reaction was quenched with H_2_O (2 mL) and diluted with ether (4 mL). The aqueous layer was extracted with Et_2_O, and the combined extracts were washed with H_2_O and brine, then dried over Na_2_SO_4_. Removal of all solvents was done *in vacuo* to yield crude an oily residue as *E/Z* isomers of **7** (0.020 g, 64%). R*_f_* = 0.33; IR (KBr, cm^−1^): *υ*: 812, 1278, 1687, 2857, 3233; ^1^H-NMR (500 MHz, CDCl_3_): δ = −0.11−0.05 (d, *J* = −30 Hz, 6H), 0.86 (s, 9H), 3.48 (s,1H), 3.91–3.92 (d, *J* = 5 Hz, 6H), 4.90–4.94 (m, *J* = 20 Hz, 1H), 5.59–5.60 (d, *J* = 5 Hz, 1H), 6.29–6.32 (d, *J* = 15 Hz, 1H), 6.37–6.48 (m, *J* = 55 Hz, 2H), 6.97 (s, 1H), 7.22 (s, 1H), 7.49 (s, 1H). ^13^C-NMR (126 MHz, CDCl_3_) δ = 153.0, 149.2, 148.8, 147.7, 141.8, 135.7, 120.7, 111.4, 110.9, 109.9, 108.1, 107.3, 69.1, 56.1, 56.0, 25.9, 18.4, −4.7, −4.7; HRMS (DART) calcd. for C_22_H_33_O_5_Si [M+H]^+^: 405.2097; not detected.

*2-(4,4-Dimethoxy-2-vinylphenyl)furan* (**8**). To a stirred solution of dry THF (36 mL) was added methyl triphenylphosphonium bromide (1.52 g, 4.32 mmol) under argon. The homogenous mixture was cooled to 0 °C followed by dropwise addition of a 2.5 M solution *n*-BuLi in hexanes (1.25 eq.). The red-orange suspension was stirred about 30 min as the color persisted under the above temp. A 0.53 mM solution of 2-furylbenzaldehyde (**6**, 0.802 g, 3.45 mmol) in THF was slowly added, and the reaction was complete at 0 °C after 10–15 mins. The reaction was quenched with sat. aq. NH_4_Cl (10 mL) and diluted with EtOAc (20 mL). The aqueous layer was extracted with EtOAc, and the combined extracts were washed with H_2_O and brine and then dried over Na_2_SO_4_. Removal of all solvents was done *in vacuo* to yield a crude solid material, which was purified by silica gel column chromatography (hexane/EtOAc, 10:1) to yield olefin **8** as a yellow oil (0.568 g, 72%). R*_f_* = 0.43; IR (KBr, cm^−1^): *υ*: 730, 909, 1266, 2254, 3007 ^1^H-NMR (500 MHz, CDCl_3_): δ = 3.93–3.95 (d, 6H, *J* = 3.3 Hz), 5.24–5.26 (dd, *J* = 5, 5 Hz, 1H), 5.59–5.62 (d, *J* = 15 Hz, 1H), 6.42–6.49 (d, *J* = 35 Hz, 2H), 7.99–7.05 (m, *J* = 30 Hz, 1H), 7.03 (s, 1H), 7.12 (s, 1H), 7.48 (s, 1H). ^13^C-NMR (126 MHz, CDCl_3_) δ = 152.64, 148.91, 141.91, 135.98, 128.73, 122.63, 114.14, 111.56, 110.39, 109.39, 56.16, 56.11; HRMS (DART) calcd. for C_14_H_15_O_3_ [M+H]^+^: 231.1021; found 231.1000.

*1-(4’,5’-Dimethoxy-2’vinylphenyl)-7-oxabicyclo[2.2.1]hepta-2,5diene-2,3-dicarboxylic acid dimethyl ester* (**9**). Dimethoxybenzylfuran **8** (343 mg, 1.49 mmol) was added to a flame dried vial in benzene (0.37mL) to make a 3 mM solution, followed by addition of DMAD (233 mg,1.64 mmol) at room temperature. The reaction stirred vigorously until everything was in solution, then placed in a preheated oil bath at 40 °C. The reaction was monitored by TLC using a 30% EtOAc in hexanes solution. Upon completion the solvents were removed *in vacuo* to afford a crude oil, which was purified by silica gel chromatography (SiO_2_, 7 g, 25% EtOAc in hexanes to yield cycloadduct **9** as an oil (0.264 g, 55%). R*_f_* = 0.21; IR (KBr, cm^−1^): *υ*: 731, 908, 1268, 2345, 3016; ^1^H-NMR (500 MHz, CDCl_3_): δ = 3.57 (s, 3H), 3.74 (s, 3H), 3.84–3.86 (d, *J* = 10 Hz, 6H), 5.14–5.16 (d, *J* = 10 Hz, 1H), 5.50–5.54 (d, *J* = 20 Hz, 1H), 5.75 (s, 1H), 6.92–7.02 (dd, *J* = 15, 15 Hz, 1H), 7.01–7.03 (d, *J* = 10 Hz, 2H), 7.27–7.28 (dd, *J* = 5 Hz, 1H), 7.47–7.48 (d, *J* = 5 Hz, 1H). ^13^C-NMR (126 MHz, CDCl_3_) δ = 164.9, 162.7, 158.8, 149.4, 149.2, 148.6, 145.2, 144.7, 134.7, 130.0, 123.6, 114.7, 110.9, 109.4, 97.9, 83.5, 55.9, 52.4; HRMS (DART) calcd. for C_20_H_21_O_7_ [M+H]^+^: 373.1287; found 373.1280.

*4’5’Dimethoxyspiro[5-oxafuryl-2,3 di(methoxycarbonyl)-4-vinyl]-1,1’[1H]indene* (**10**). To a solution of oxabicyclic ether **9** (177 mg, 0.475 mmol) in dry dichloromethane (47.5 mL) was added a solution of Grubbs 2nd generation catalyst **G-II** (40 mg, 0.0475 mmol) in 1 mL CH_2_Cl_2_ at room temperature. The reaction was stirred vigorously at room temperature then placed into a preheated oil bath at 35 °C. The reaction was monitored by ^1^H-NMR. After 24 hrs of heating, the reaction was removed from heat and concentrated to a crude brown oil. Purification of the residue by flash chromatography using 25% EtOAc in hexanes afforded pure spirocyclic compound **10** (0.048g, 50%). R*_f_* = 0.21; IR (KBr, cm^−1^): *υ*: 732, 907, 1255, 2253, 2955; ^1^H-NMR (500 MHz, CDCl_3_): δ = 3.56 (s, 3H), 3.81 (s, 3H), 3.87–3.89 (d, *J* = 10 Hz, 6H), 5.28–5.30 (d, *J* = 20 Hz, 1H), 5.48–5.51 (d, *J* = 15, 1H), 5.76–5.78 (s,1H) 5.99–6.04 (m, 1H), 6.16–6.17 (d, *J* = 5.5 Hz, 1H), 6.61–6.62 (d, *J* = 5.5 Hz, 1H), 6.76 (s, 1H), 6.89 (s, 1H). ^13^C-NMR (126 MHz, CDCl_3_) δ = 163.1, 162.3, 150.3, 148.4, 142.9, 136.7, 136.6, 135.7, 133.7, 128.8, 127.0, 118.1, 107.8, 105.8, 99.38 86.4, 56.5, 56.6, 52.6; HRMS (DART) calcd. for C_20_H_21_O_7_ [M+H]^+^: 373.1287; found 373.1283.

*1-(2’-Formyl-4’,5’dimethoxyphenyl)-7-oxabicyclo[2.2.1]hepta-2,5-diene-2,3-dicarboxylic acid dimethyl ester* (**12**). Dimethoxyfurylbenzaldehyde **6** (343 mg,1.49 mmol) in benzene (0.37 mL) was added to a flame dried vial to make a 3 mM solution, followed by addition of DMAD (233 mg, 1.64 mmol) at room temperature. The reaction was stirred vigorously until everything was in solution, then added to a preheated oil bath at 50 °C. The reaction was monitored by TLC using a 30% EtOAc solution in hexanes as eluent. Upon completion the solvents were removed *in vacuo* to afford a crude oil, which was purified by silica gel chromatography (SiO_2_, 7 g, 25% EtOAc in hexanes) to yield cycloadduct **12** as an oil. Diastereomers were obtained, which could be separated via column chromatography (0.202 g, 50% of desired product **12**). R*_f_* = 0.085; R*_f_* = 0.17; m.p. = 175 °C; IR (KBr, cm^−1^): *υ*: 730, 912, 1291, 1684, 3024; ^1^H NMR (500 MHz, CDCl_3_): δ = 3.63 (s, 3H), 3.81 (s, 3H), 3.97–3.98 (d, *J* = 5 Hz, 6H), 5.76 (s, 1H), 7.21 (s, 1H), 7.25 (s, 2H), 7.30–7.33 (dd, *J* = 5, 5 Hz, 1H), 7.45 (s, 1H), 10.03 (s, 1H). ^13^C-NMR (126 MHz, CDCl_3_) δ = 190.9, 164.1, 162.7, 156.8, 153.5, 148.9, 148.3, 144.9, 143.3, 129.9, 126.9, 114.6, 110.3, 96.6, 82.6, 56.2, 56.1, 52.3, 52.2; HRMS (DART) calcd. for C_19_H_18_O_8_: [M+H]^+^: 375.1080; found 375.1069.

*4’,5’-Dimethoxy-2-(2,3,4,4-tetrachloro-8-oxabicyclo[3.2.1]octa-2,6-dien-1-yl-benzaldehyde* (**14**). Dimethoxyfurylbenzaldehyde **6** (0.237 mmol) was dissolved in benzene to make a 2.97 M solution at room temperature, followed by addition of tetrachlorocyclopropene (1 eq.). The homogeneous mixture was stirred vigorously, heated to 55 °C and stirred for 3–4 days. The reaction was monitored by TLC using a 25% EtOAc in hexanes solution. Upon completion the solvents were removed *in vacuo* to afford a crude solid, which was purified by flash chromatography (SiO_2_, 9 g, 25% EtOAc in hexanes) to yield a yellow solid of **14** (0.42g, 68%). R*_f_* = 0.67; m.p. = 175 °C; IR (KBr, cm^−1^): *υ*: 733, 907, 1277, 1516, 2938; ^1^H-NMR (500 MHz, CDCl_3_): δ = 3.98–4.07 (d, *J* = 43 Hz, 6H), 5.46 (s, 1H), 7.00 (s, 1H), 7.30 (s, 1H), 7.59 (s, 1H), 10.23 (s, 1H). ^13^C-NMR (126 MHz, CDCl_3_) δ = 190.1, 153.0, 150.2, 144.7, 138.7, 129.4, 127.9, 122.5, 112.6, 110.3, 94.2, 82.6, 75.2, 64.1, 63.3, 56.3, 56.2; HRMS (DART) calcd. for C_16_H_13_Cl_4_O_4_ [M+H]^+^: 410.9540; found 410.9540.

*2,3,4,4,Tetrachloro-1-(4’,5’dimethoxy-2’-vinylphenyl-8-oxabicyclo[3.2.1]octa-2,6-diene* (**15**). Cyclo-adduct **14** (0.237 mmol) was olefinated as mentioned above to give crude compound **15** which was purified using flash chromatography on silica gel (hexane/EtOAc, 20:1) to yield a yellow solid (0.57 g, 45%). R*_f_* = 0.71; IR (KBr, cm^−1^): *υ*: 731, 908, 2252, 2941, 3014; ^1^H-NMR (500 MHz, CDCl_3_): δ = 3.95–3.99 (d, *J* = 16 Hz, 6H), 5.18–5.21 (d, *J* = 12Hz, 1H), 5.39 (s, 1H), 5.54–5.57 (d, *J* = 12 Hz, 1H), 6.88–7.00 (dd, *J* = 4 Hz, dd, *J* = 8 Hz, 1H), 6.93 (s, 1H), 6.96 (s, 1H), 7.09 (s, 1H), 7.19 (s, 1H). ^13^C-NMR (126 MHz, CDCl_3_) δ = 150.3, 148.4, 145.2, 137.3, 135.1, 132.7, 120.8, 115.0, 113.5, 110.0, 95.6, 82.4, 75.5, 64.7, 63.6, 56.2, 56.1; HRMS (DART) calcd. for C_17_H_15_Cl_4_O_3_ [M+H]^+^: 408.9747; found 408.9767.

*4’5’Dimethoxyspiro[6-oxapyrano-2,3,4,4 tetrachloro-5-vinyl]-1,1’[1H]indene* (**16**). Purification of the crude residue **16** by flash chromatography using 25% EtOAc in hexanes afforded pure spiro-fused pyran. Yield: 0.048 g (48%). R*_f_* = 0.30; IR (KBr, cm^−1^): *υ*: 733, 912, 2324, 2861, 2943; ^1^H-NMR (500 MHz, CDCl_3_): δ = 3.90–3.91 (d, *J* = 5 Hz, 6H), 5.07–5.08 (d, *J* = 5 Hz, 1H), 5.45–5.47 (d, *J* = 10 Hz, 1H), 5.52 (s, 1H) 6.12–6.13 (d, *J* = 5 Hz, 1H), 6.22–6.28 (m, *J* = 30 Hz, 1H), 6.59–6.60 (d, *J* = 5 Hz, 1H), 6.81(s, 1H), 7.68 (s, 1H). ^13^C-NMR (126 MHz, CDCl_3_) δ = 150.9, 148.5, 144.9, 137.8, 137.3, 134.3, 132.6, 131.3, 124.9, 121.2, 111.8, 106.5, 84.0, 70.20, 59.53, 56.6, 56.4; HRMS (DART) calcd. for C_17_H_15_Cl_4_O_3_ [M+H]^+^: 408.9747; found 408.9772. 

## 4. Conclusions

The highly oxygenated oxaspirocyclic core of phelligridins G and E presents an interesting synthetic challenge, while the potential anticancer and antioxidant properties of the molecules make them compelling targets for study. In this work, we investigated two potential furan-based routes to the oxaspiro core of these compounds. Initial attempts to affect an electrochemical oxidative cyclization were complicated by facile over-oxidation of the products. An alternative route involving a challenging Diels-Alder cycloaddition with a highly substituted 2-styrylfuran, followed by a tandem ring-opening/ring-closing (domino) metathesis reaction proved successful. It was also possible to extend this strategy to the higher homolog by using a formal [4+3] cycloaddition reaction with tetrachlorocyclopropene. Current efforts towards the synthesis of phelligridin G based on this strategy are ongoing.

## Supplementary Materials

Supplementary materials can be accessed at: http://www.mdpi.com/1420-3049/18/2/2438/s1.
