# Supplementary figures and images for: The Tandem Ring Opening/Ring Closing Metathesis Route to Oxaspirocycles: An Approach to Phelligridin G
Source: Molecules. 2013 Feb 21;18(2):2438–48. doi: 10.3390/molecules18022438 (PMC6269797; doi:10.3390/molecules18022438)

## Supplementary Materials

$^1\text{H}$ -NMR and  $^{13}\text{C}$ -NMR spectra.

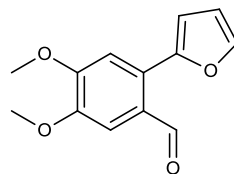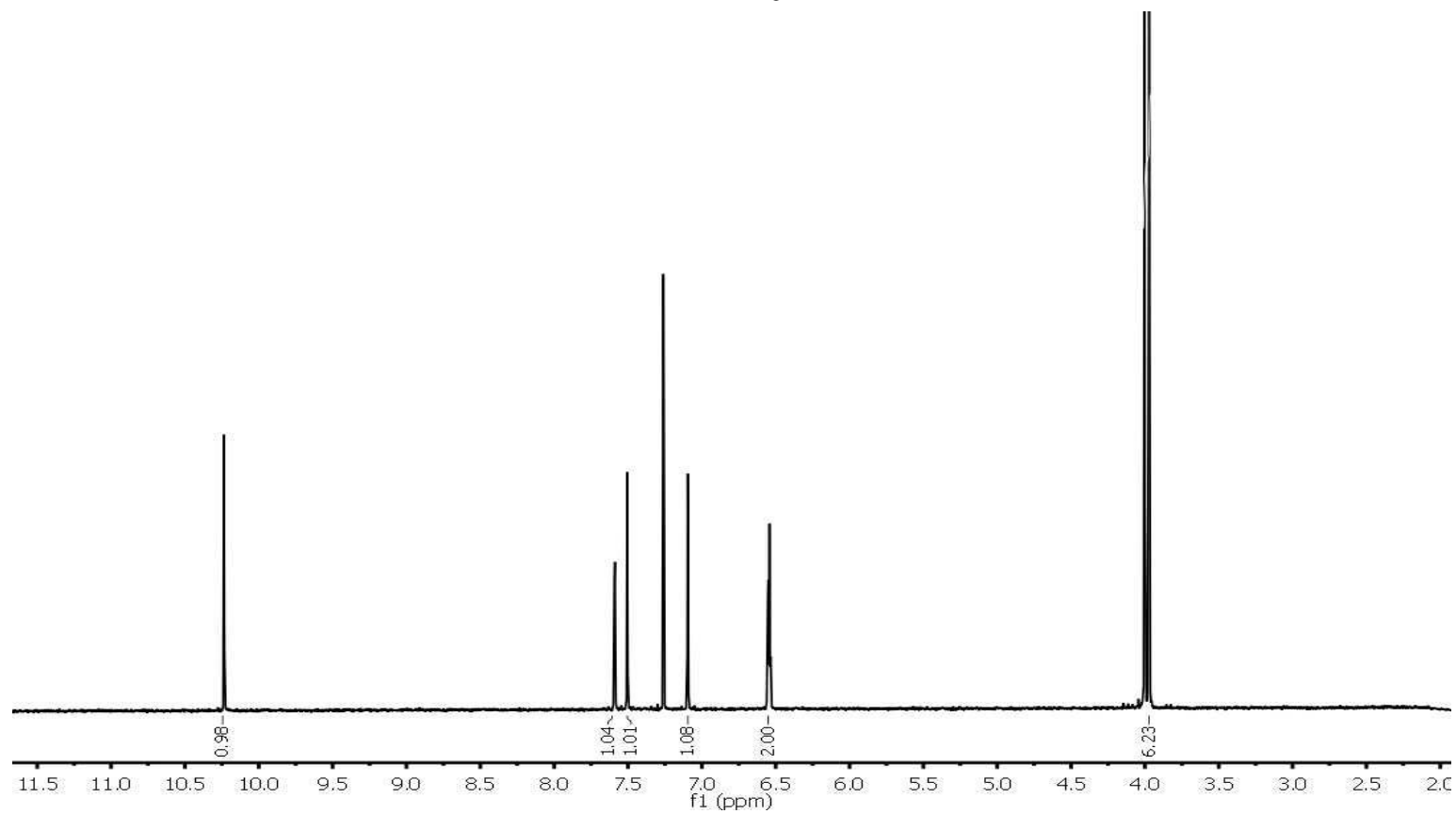

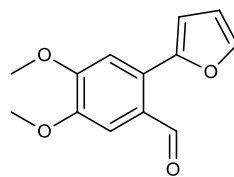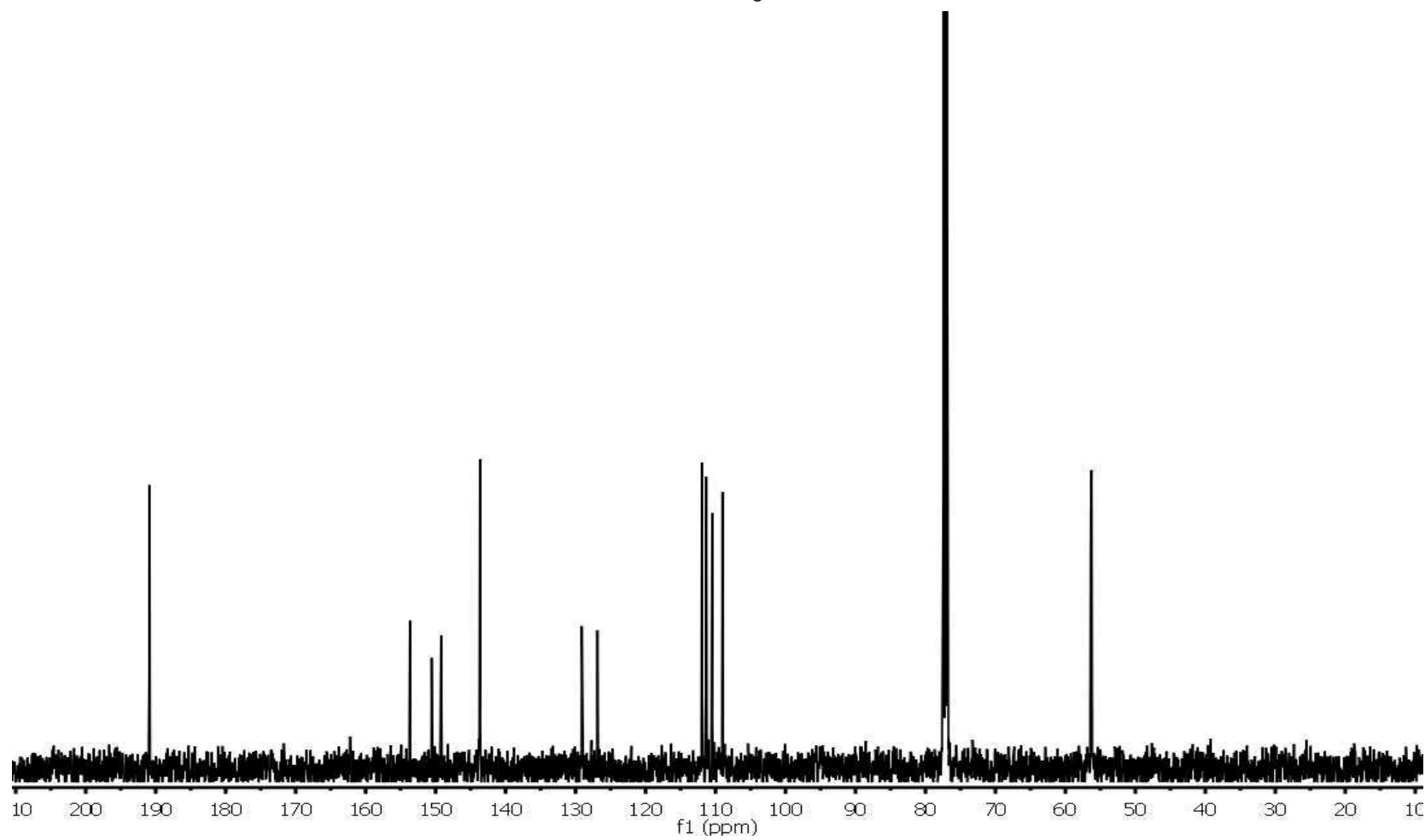

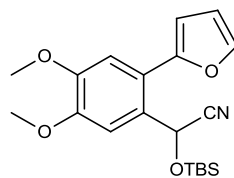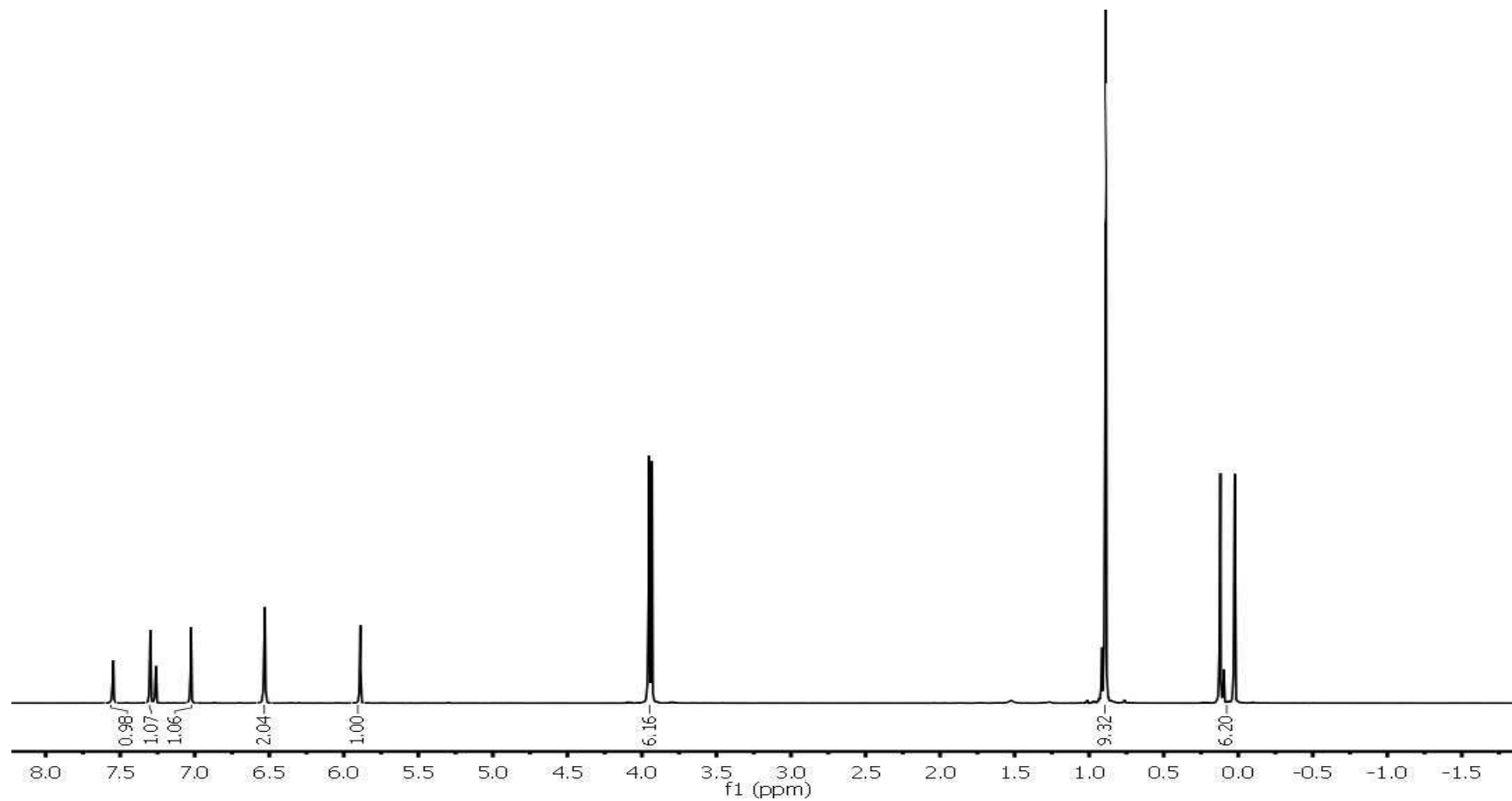

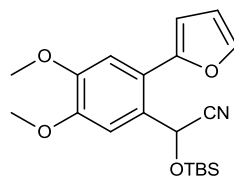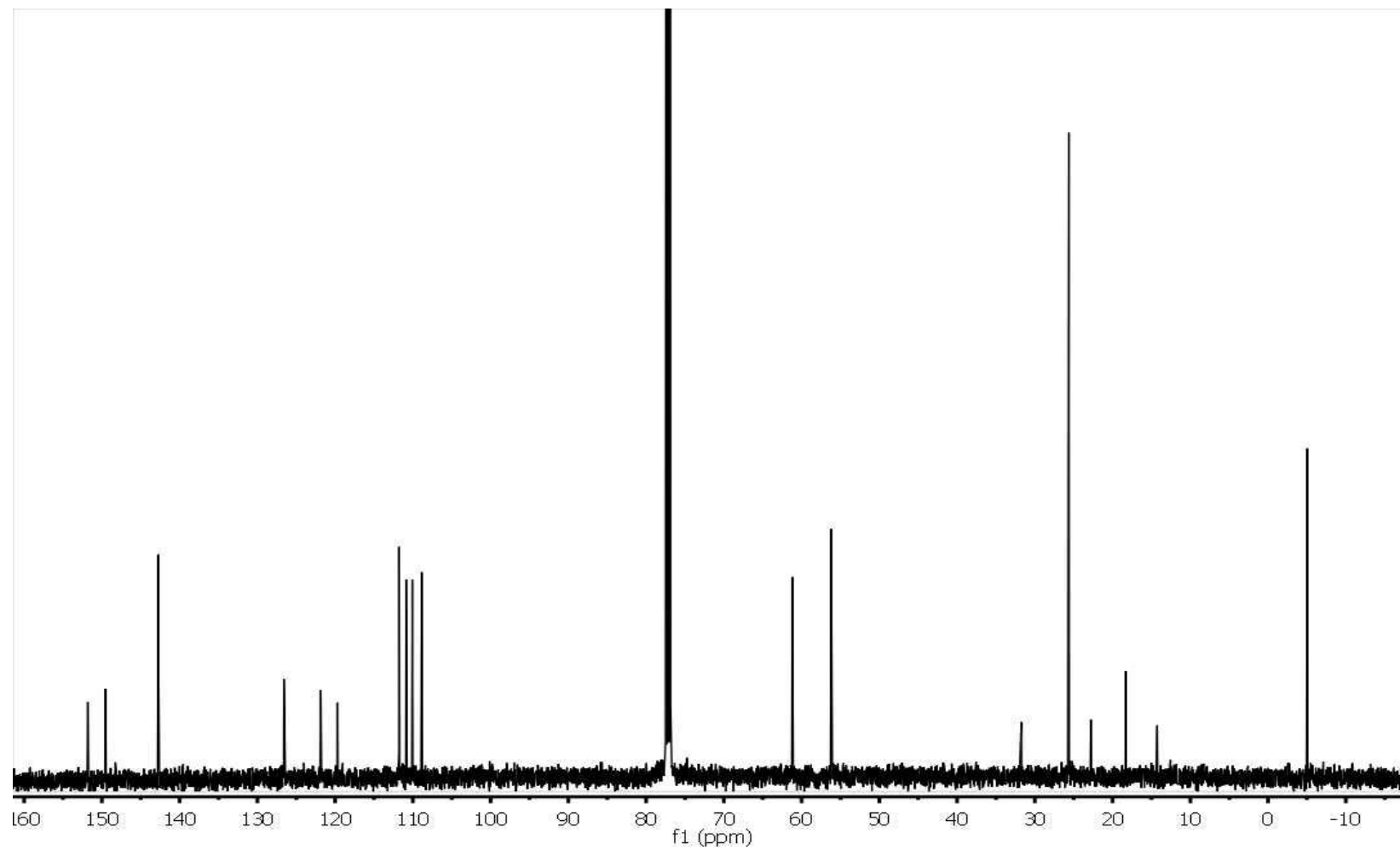

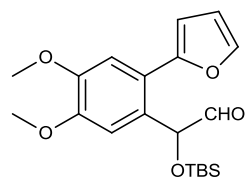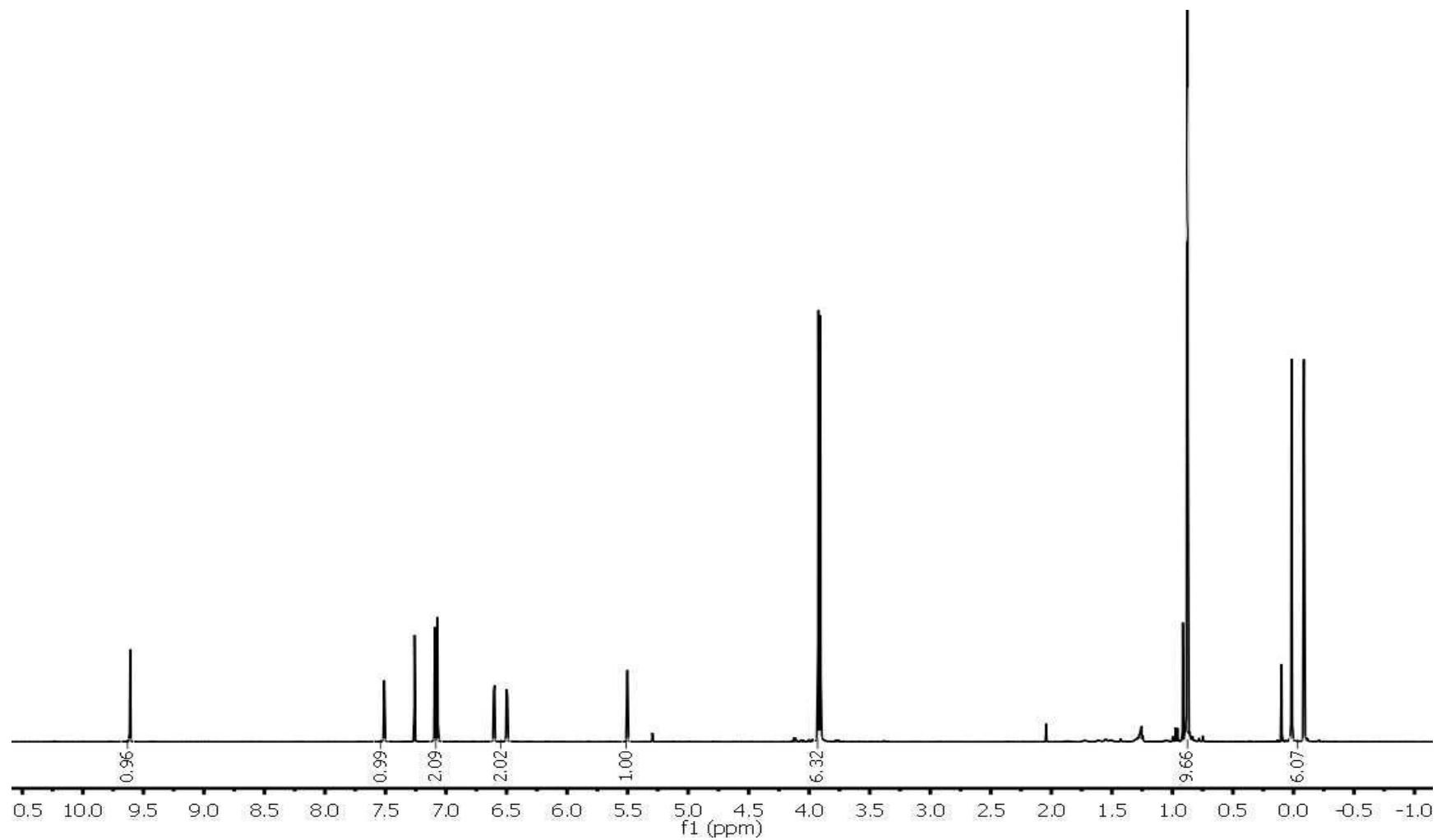

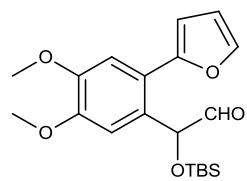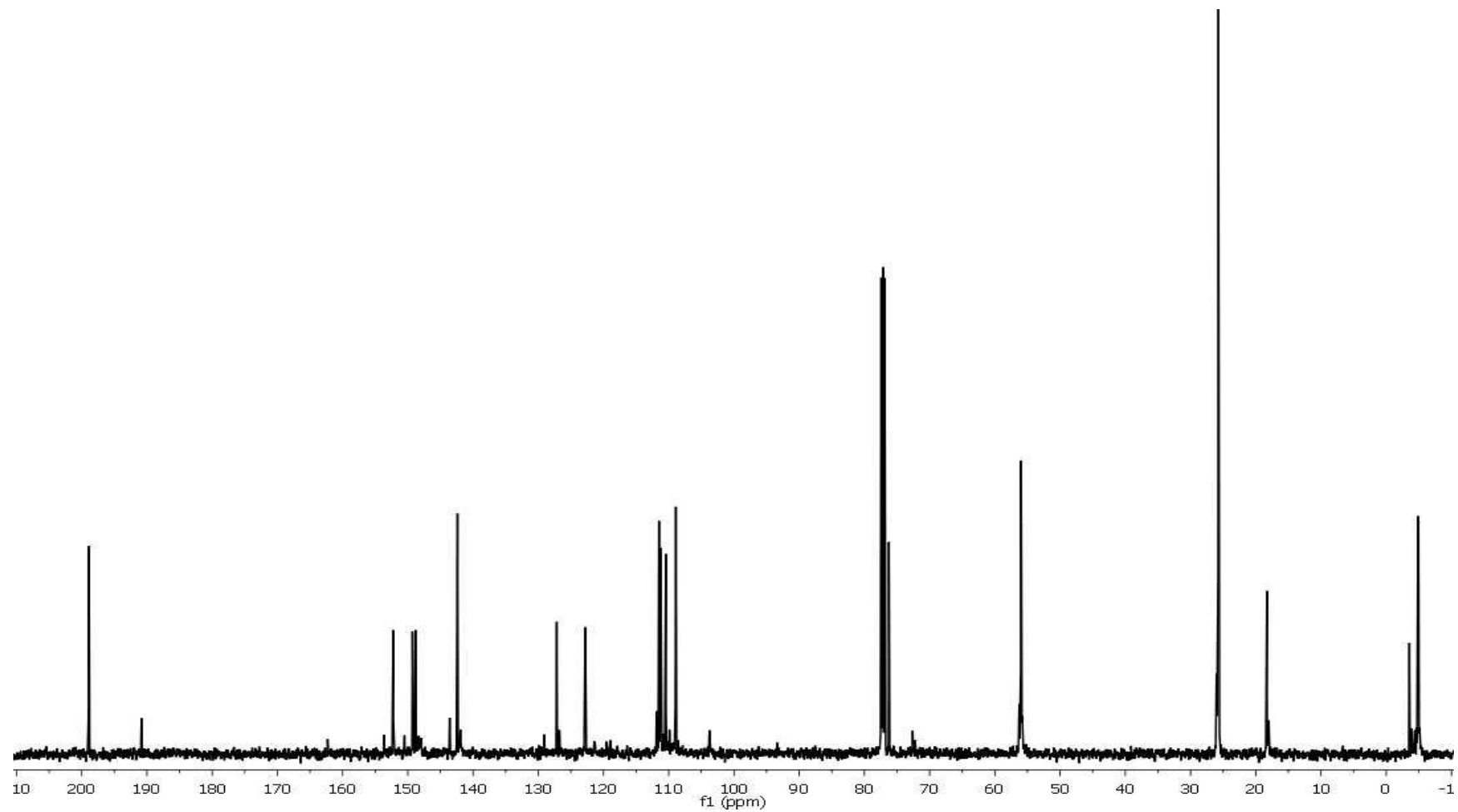

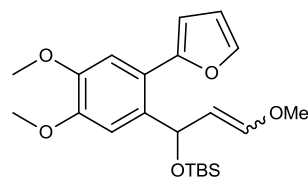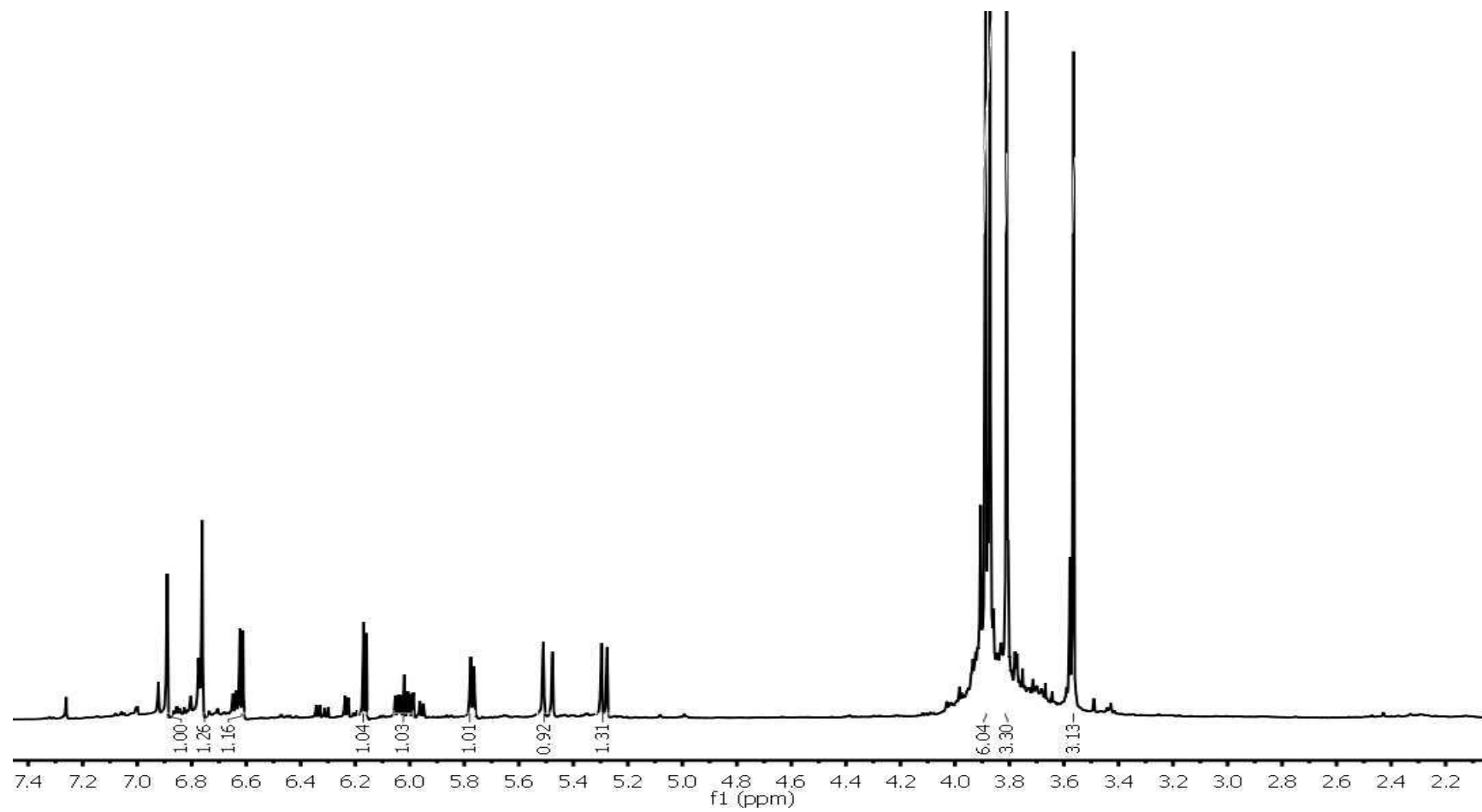

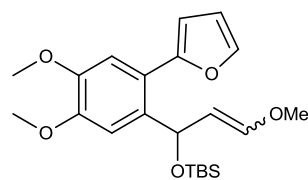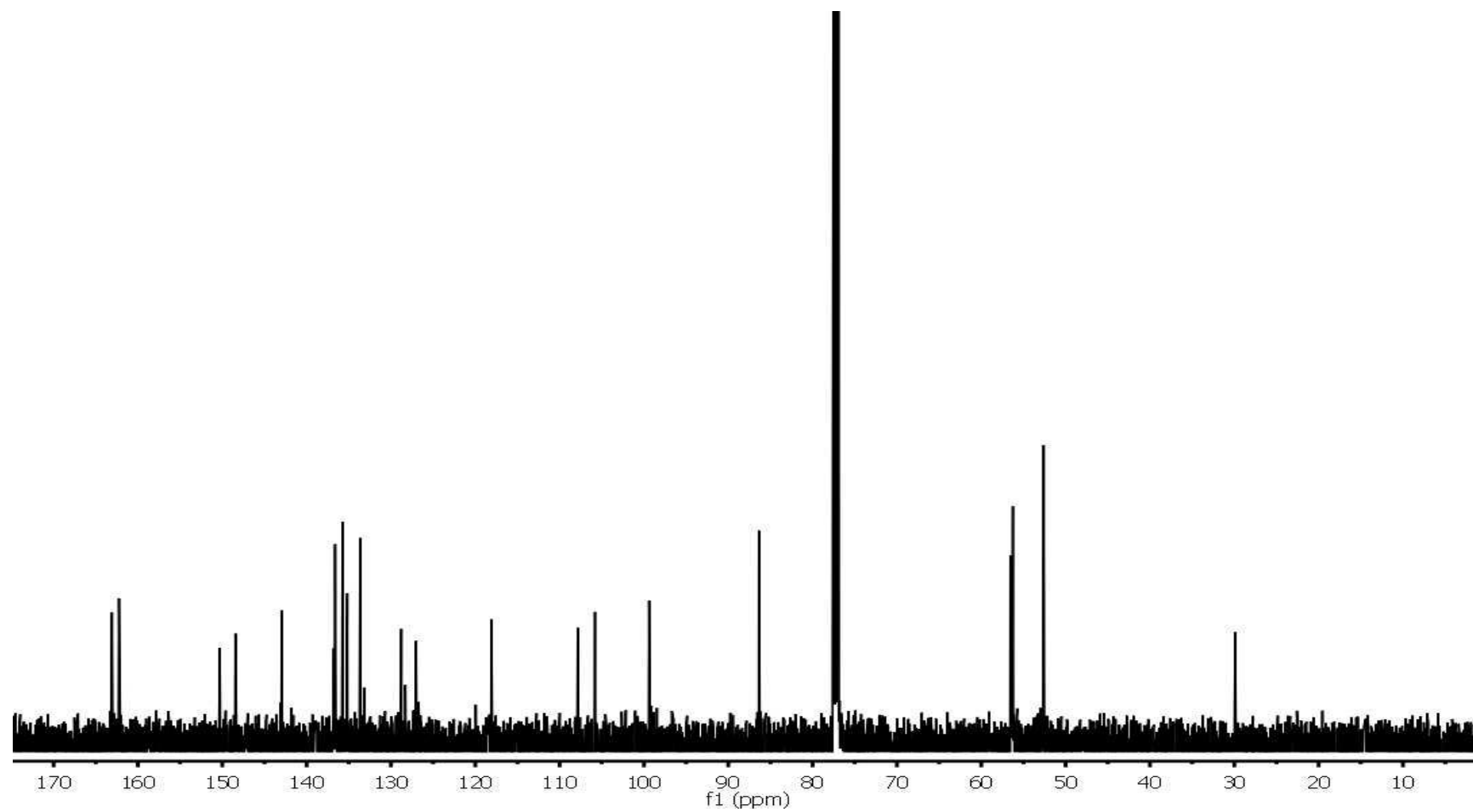

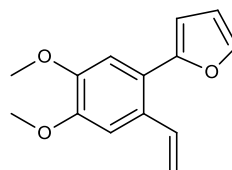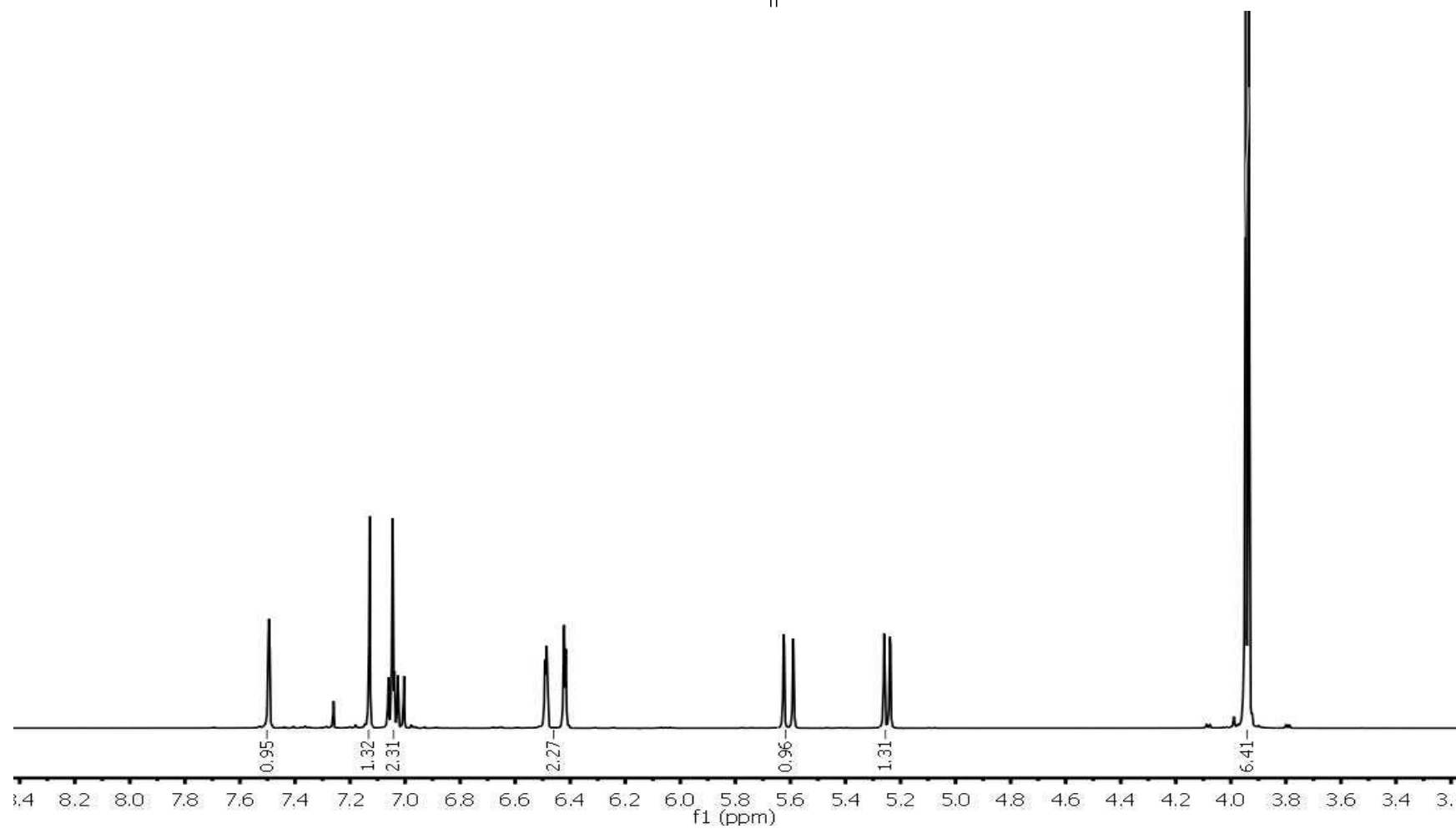

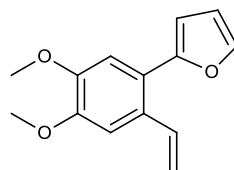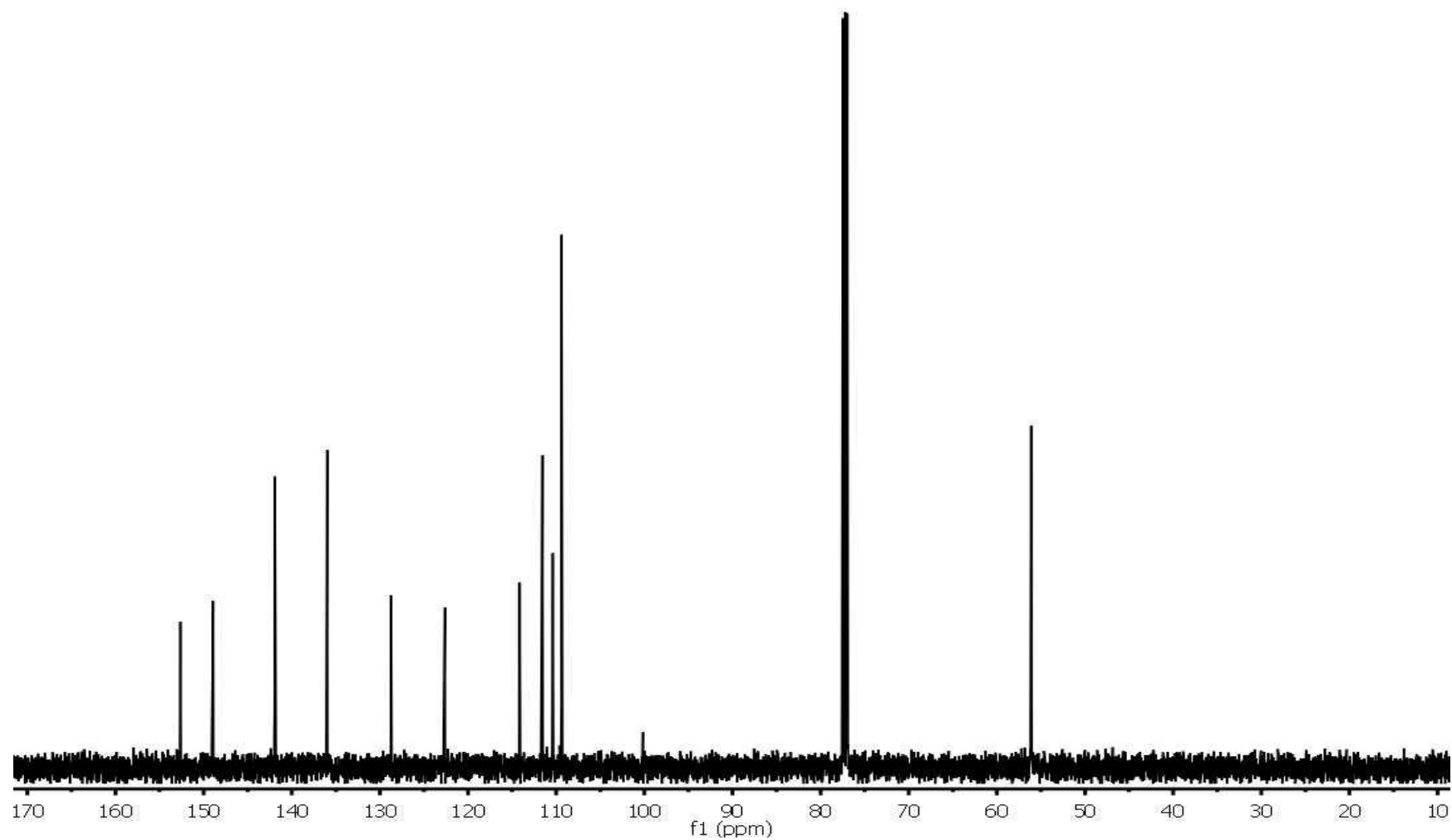

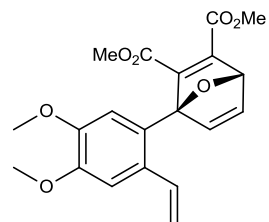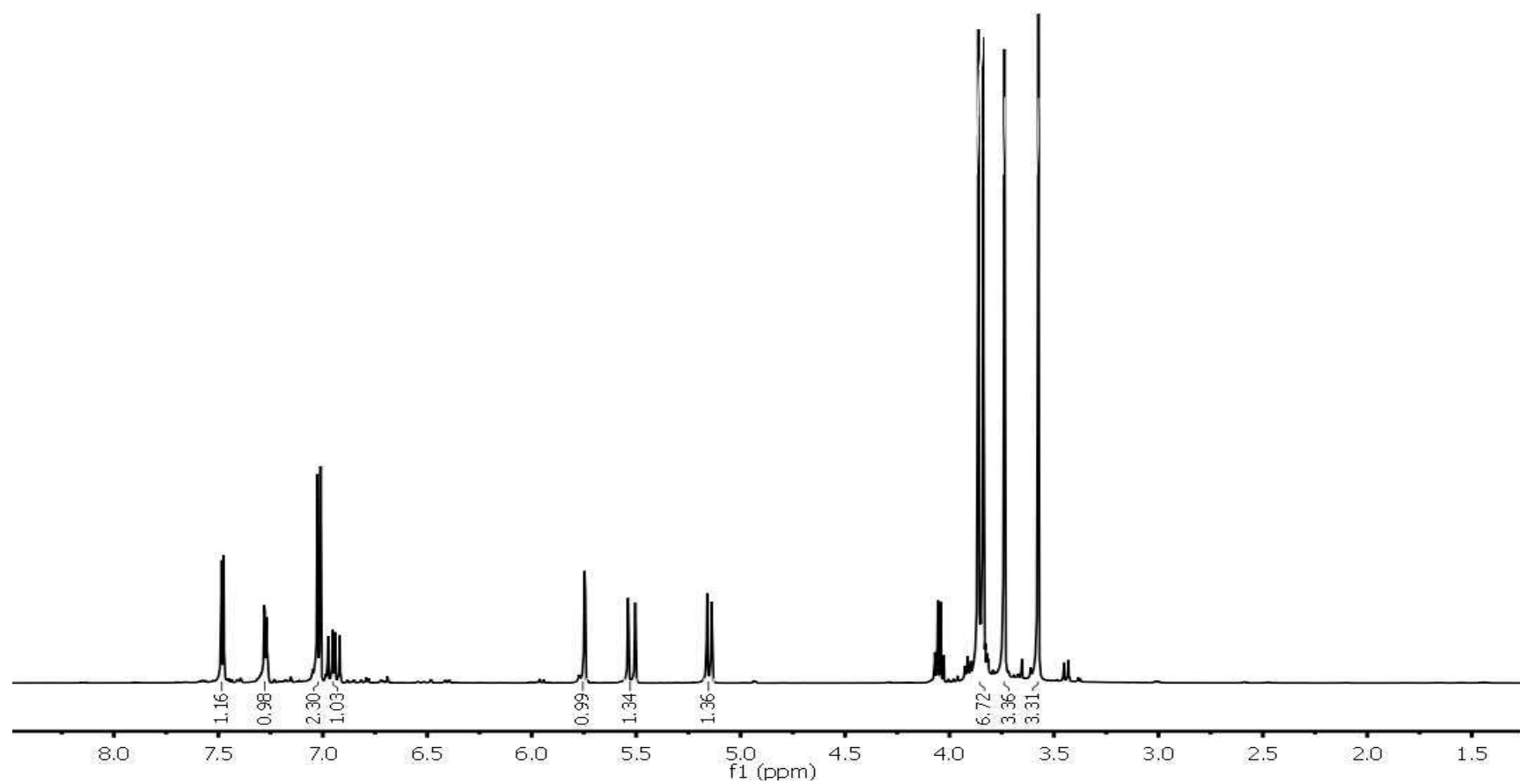

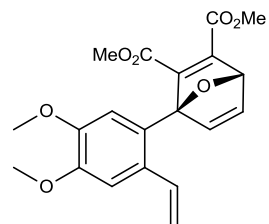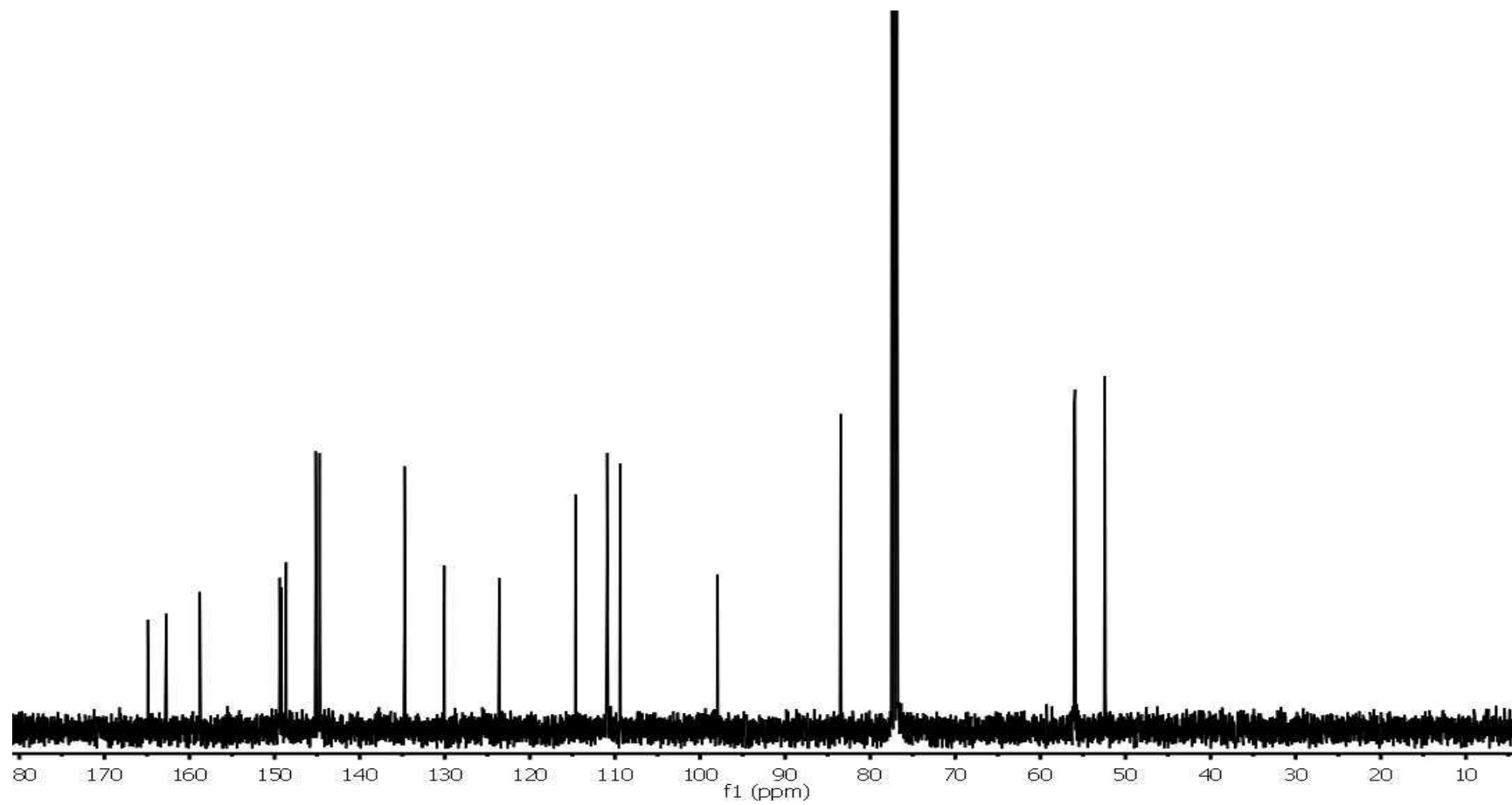

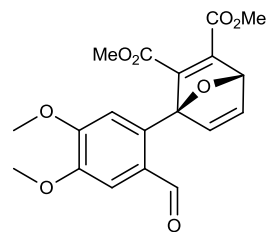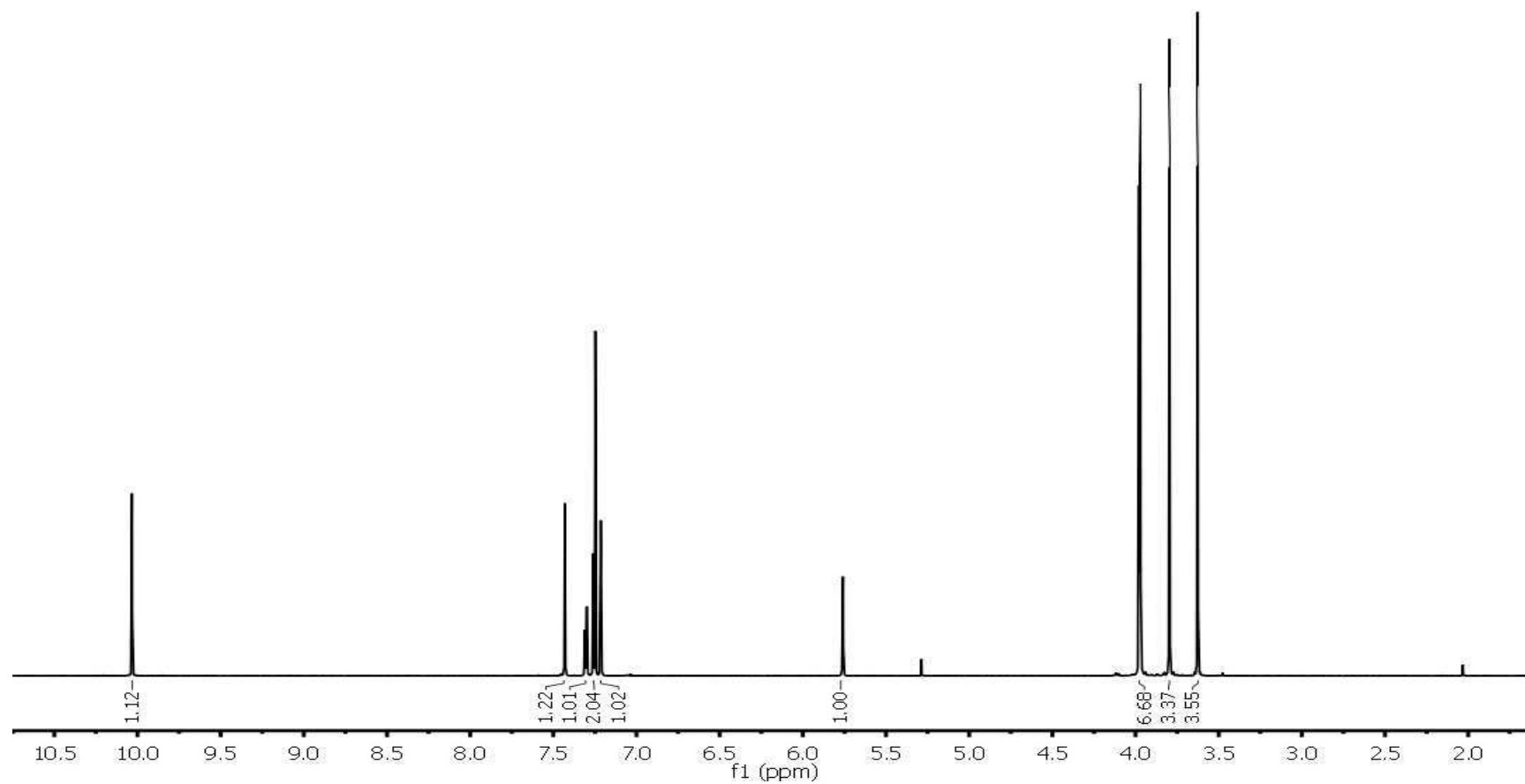

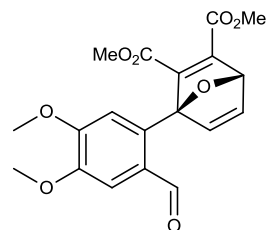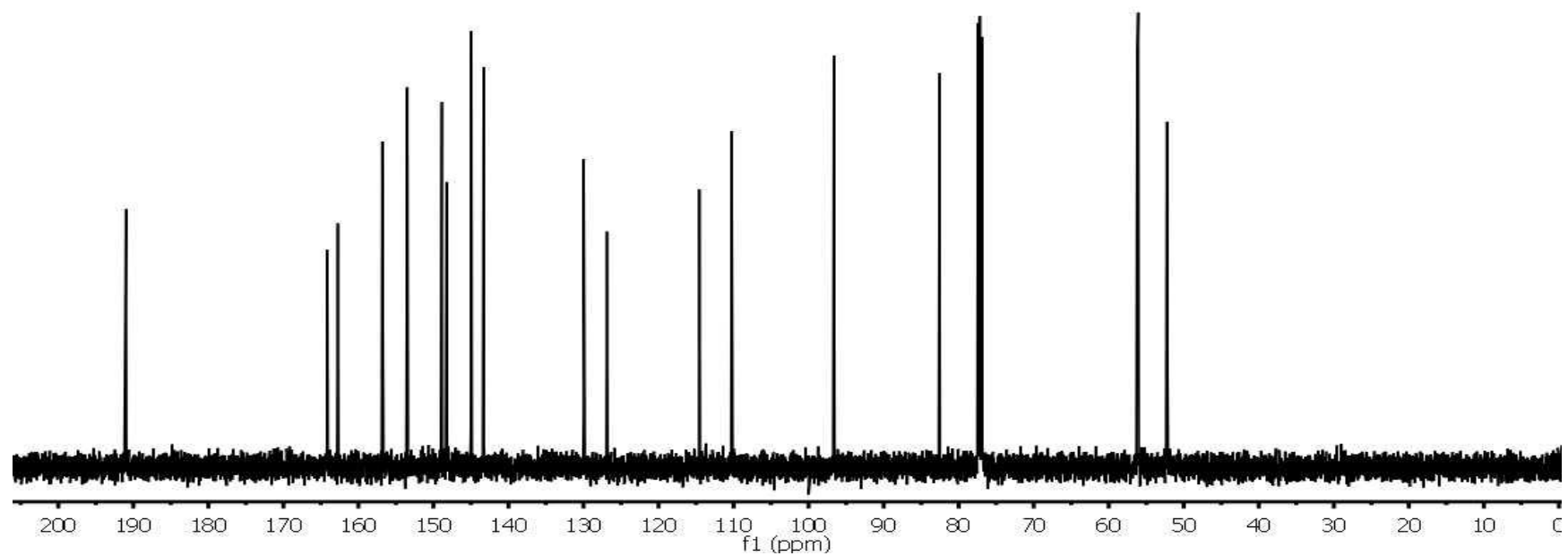

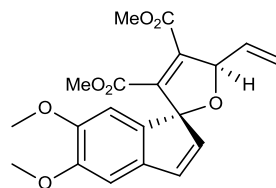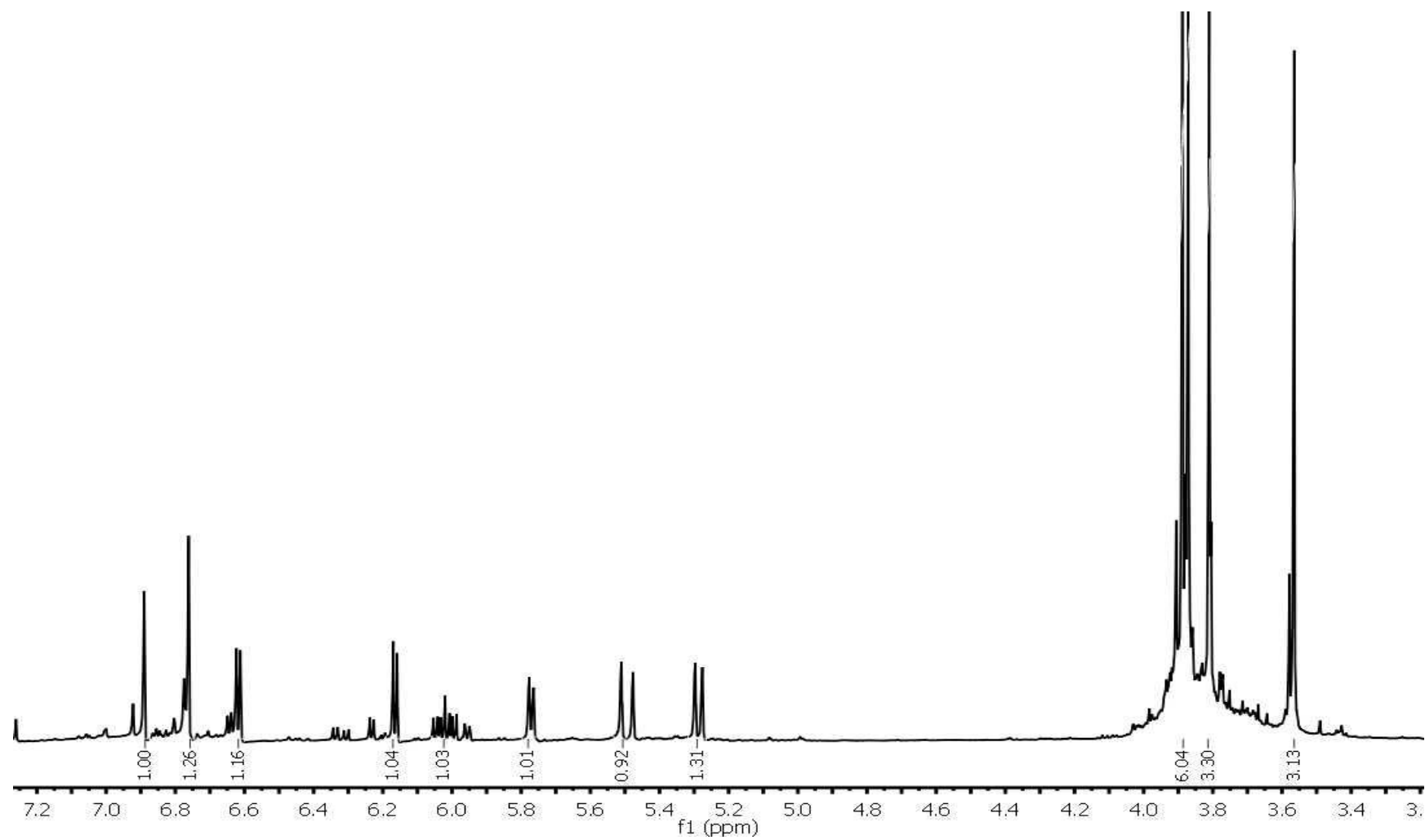

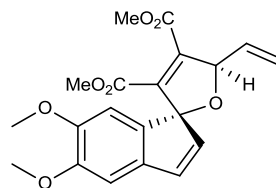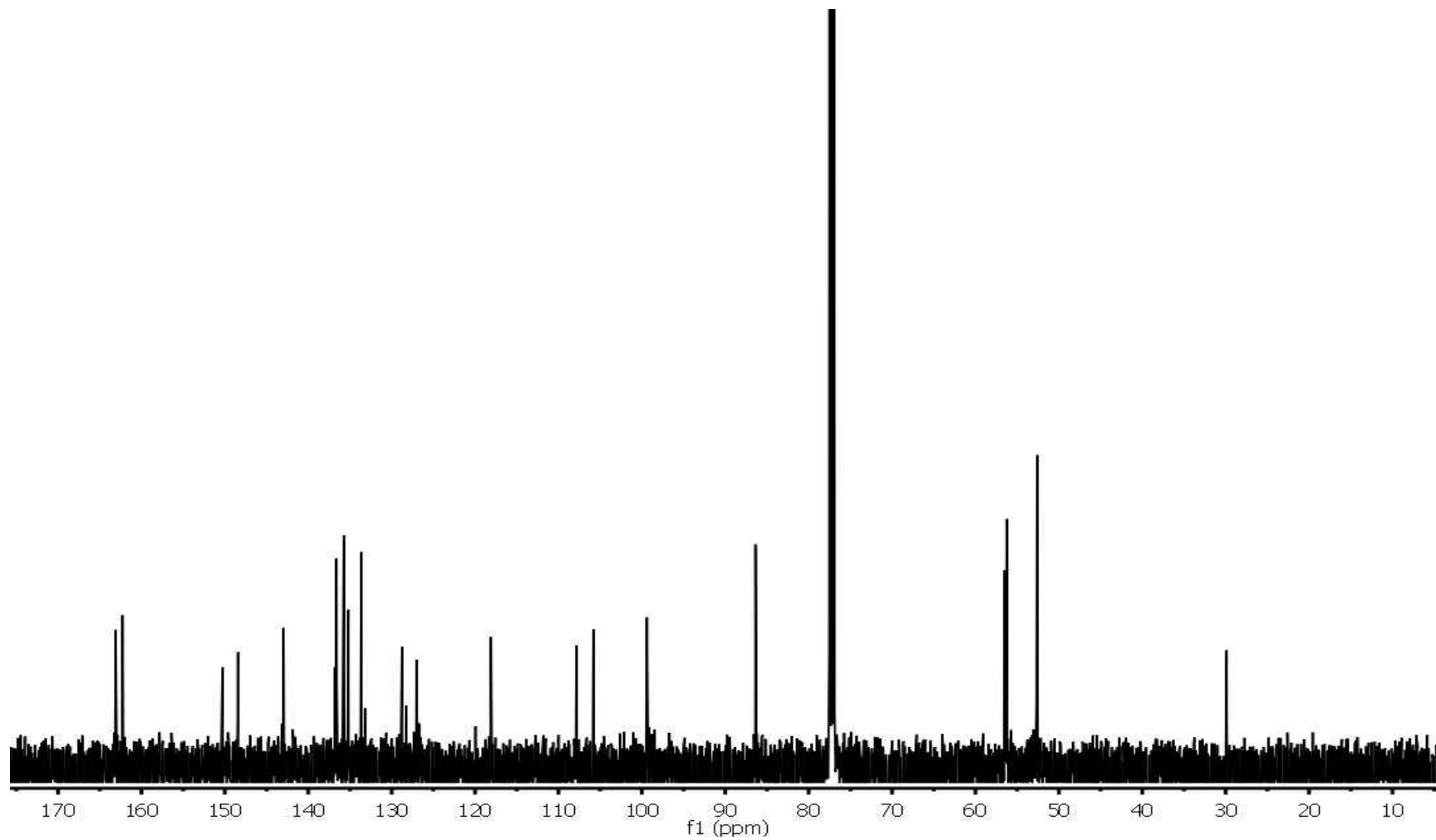

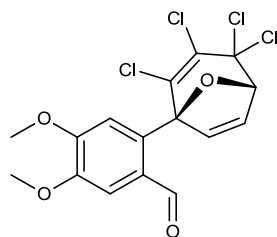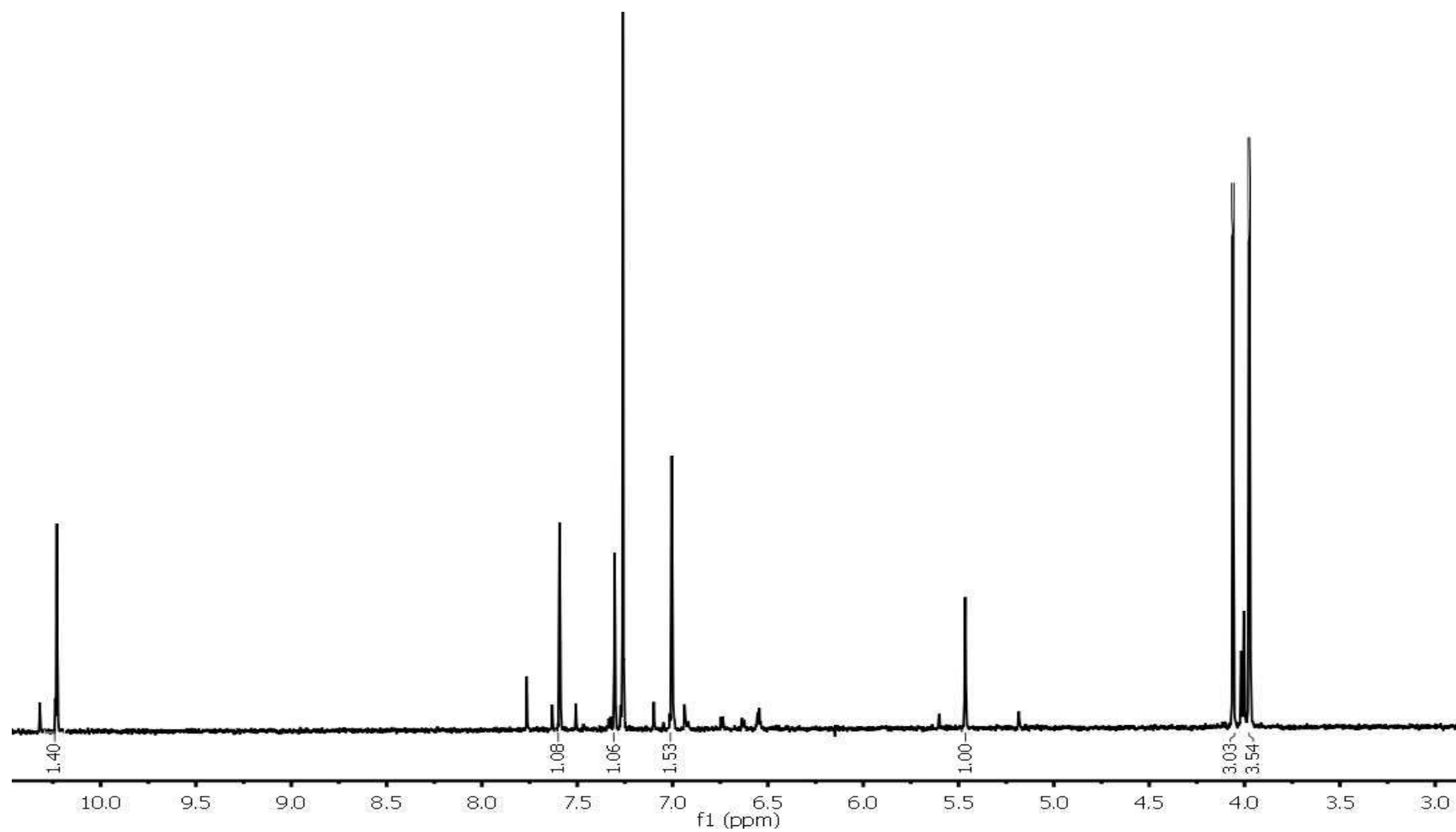

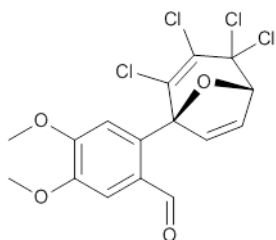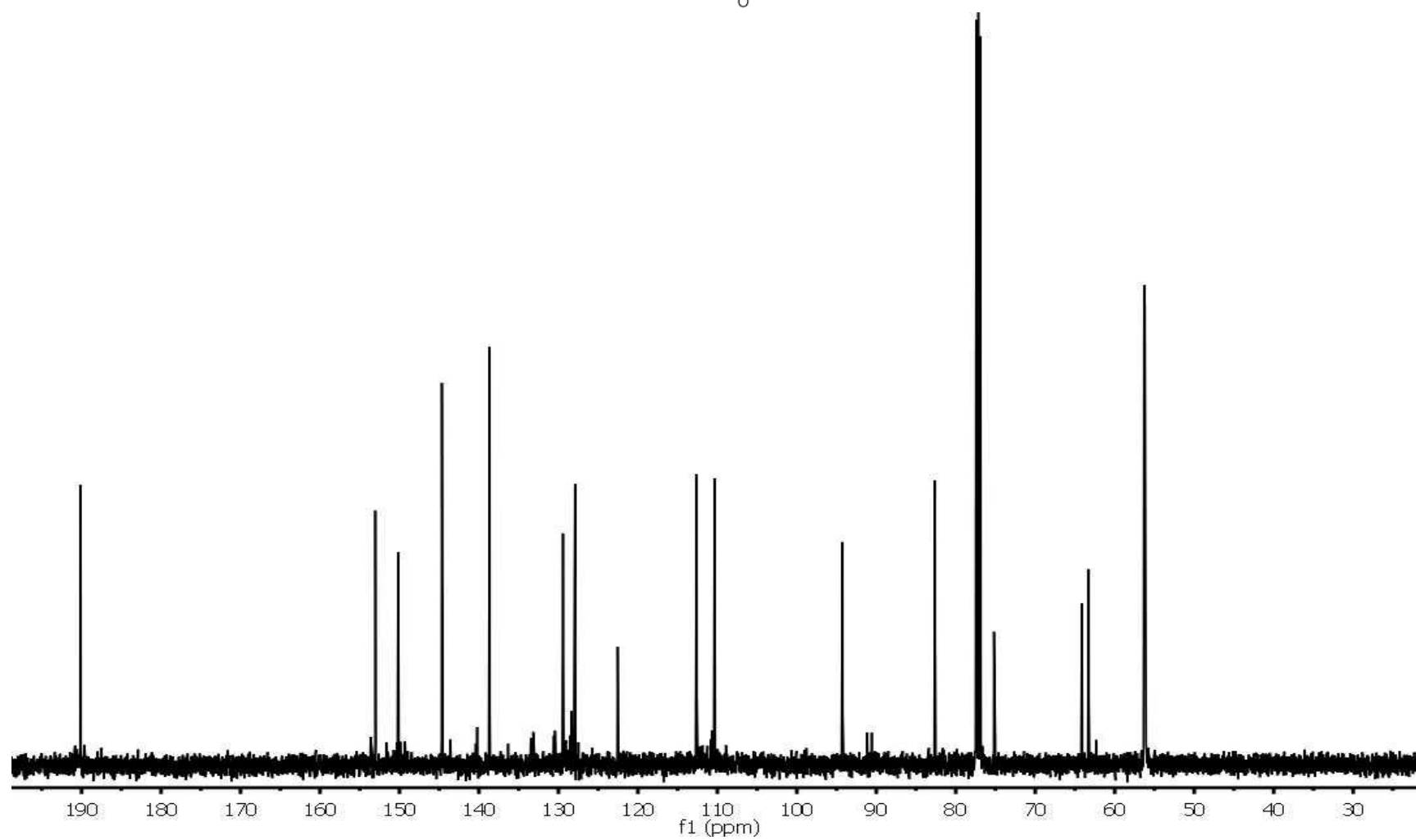

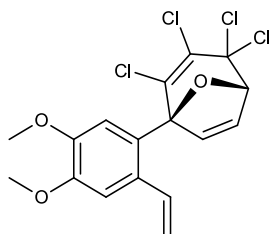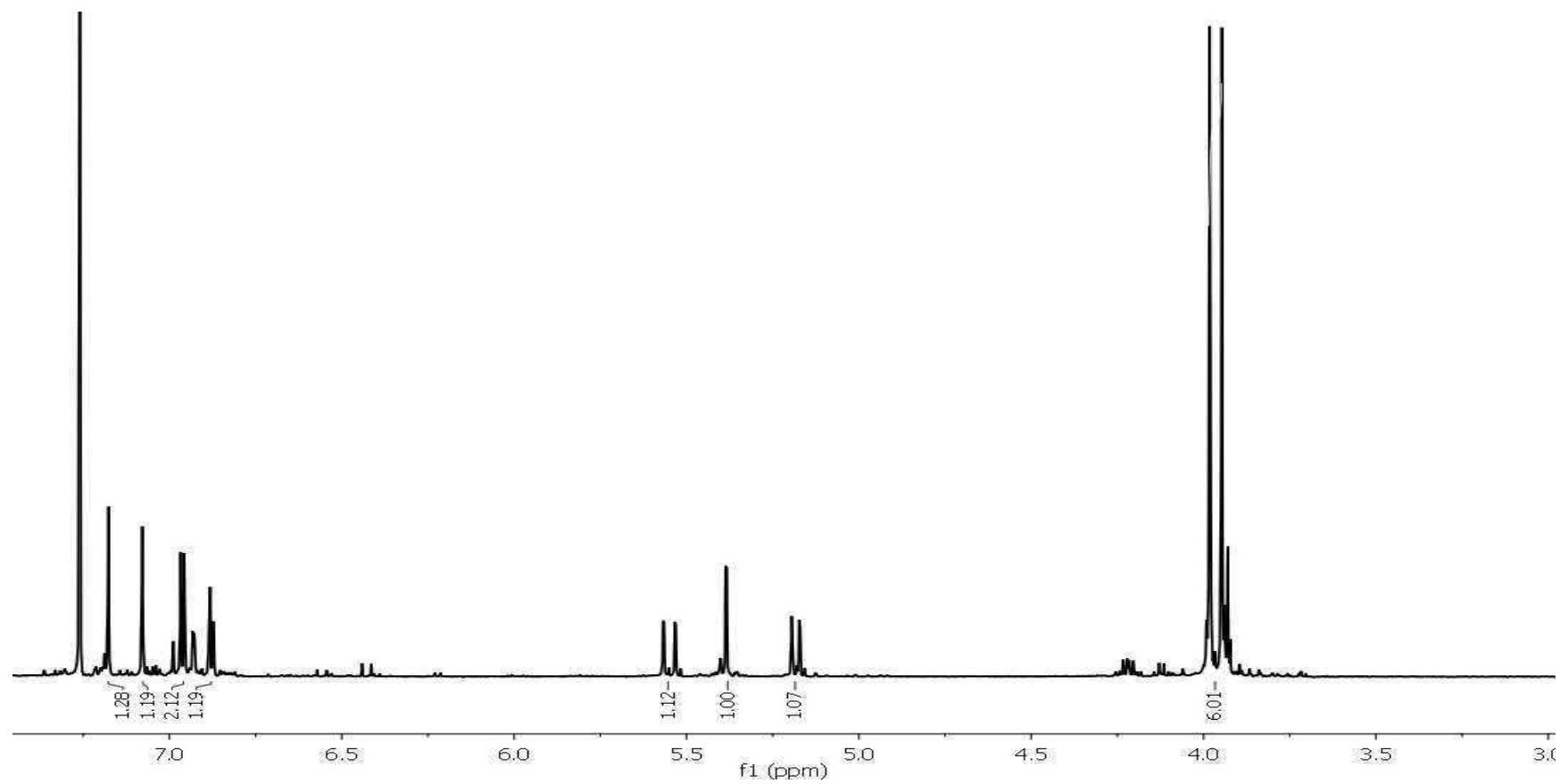

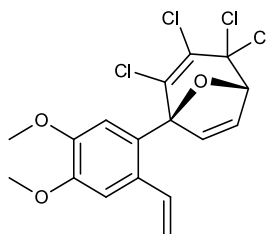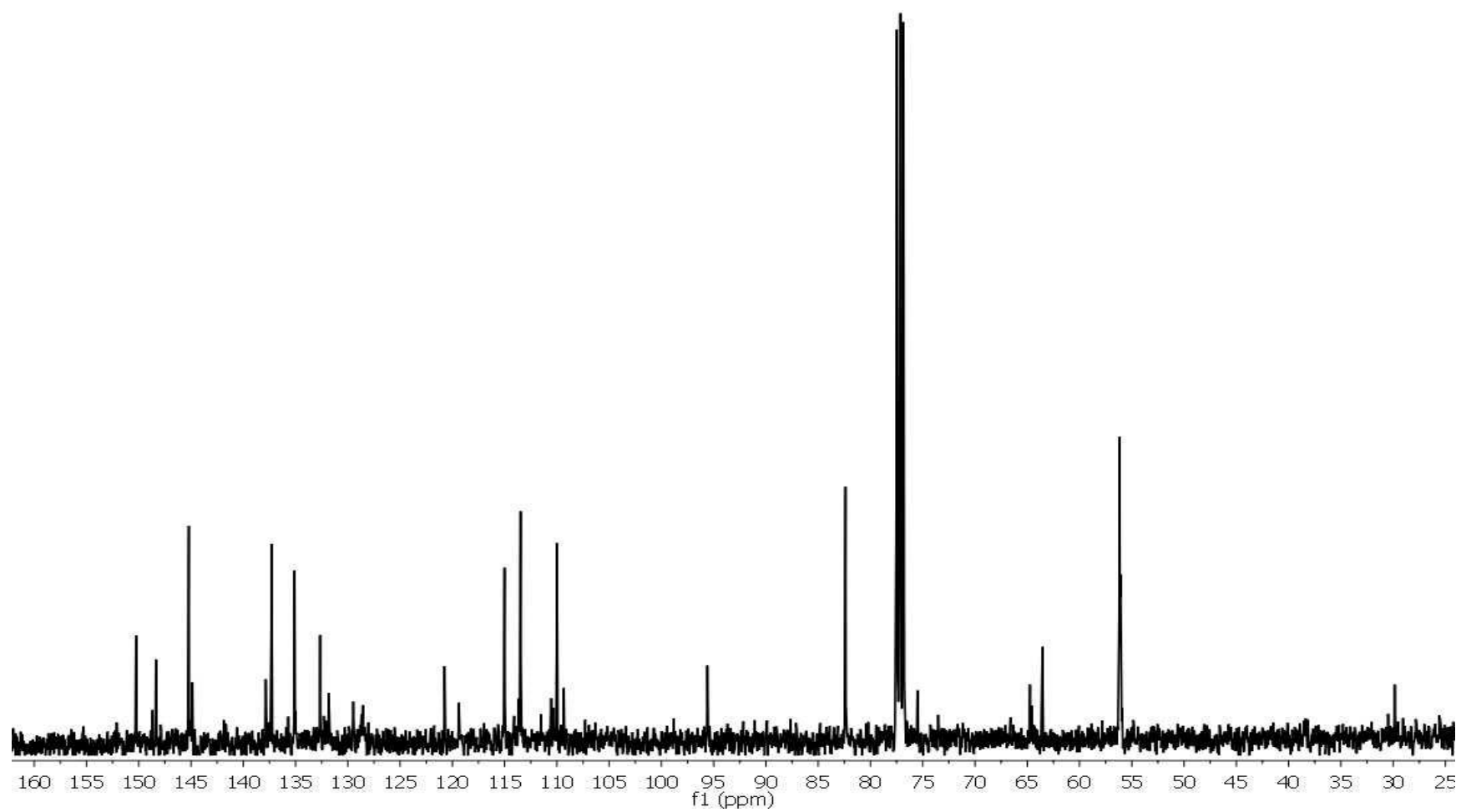

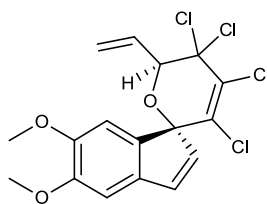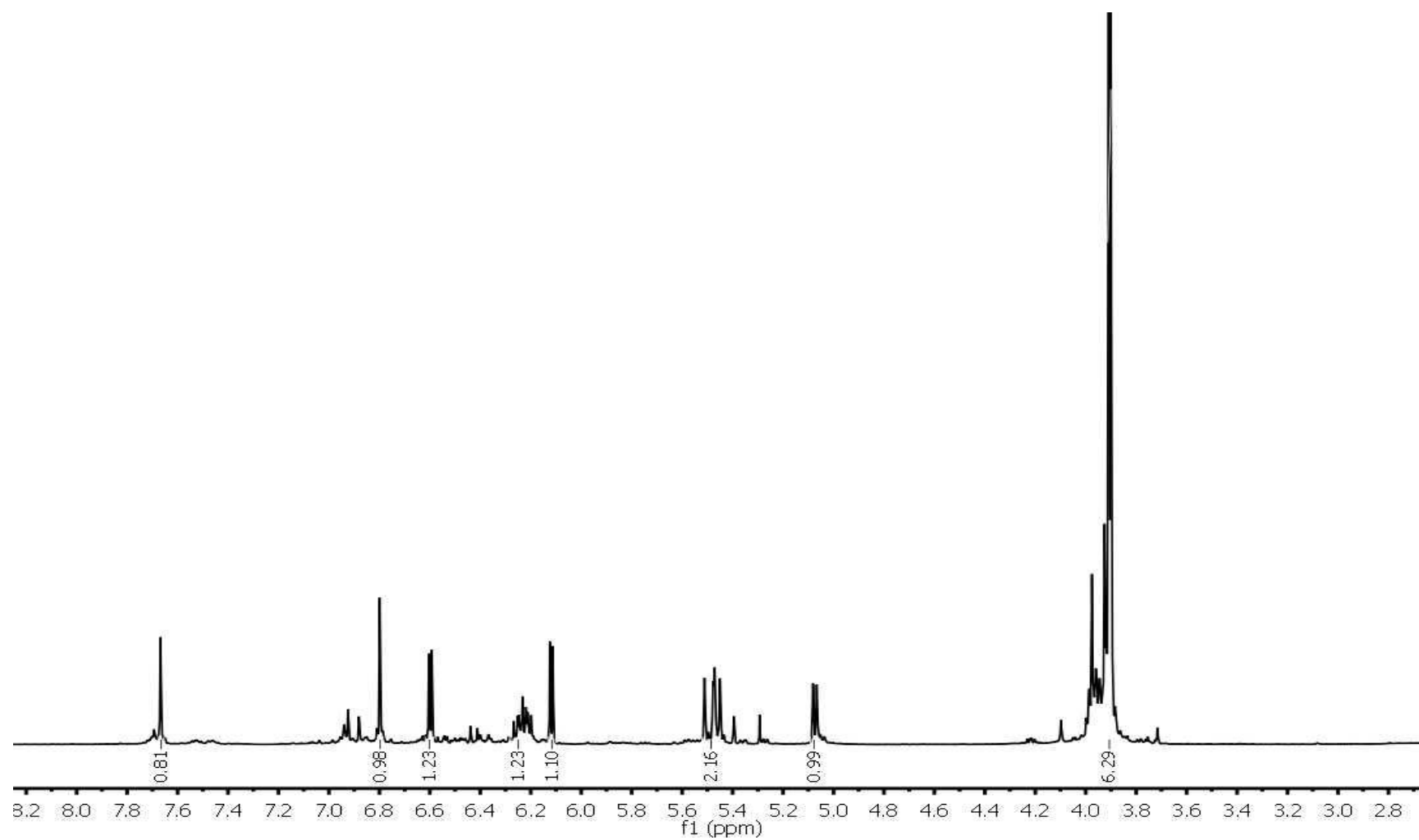

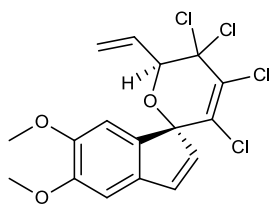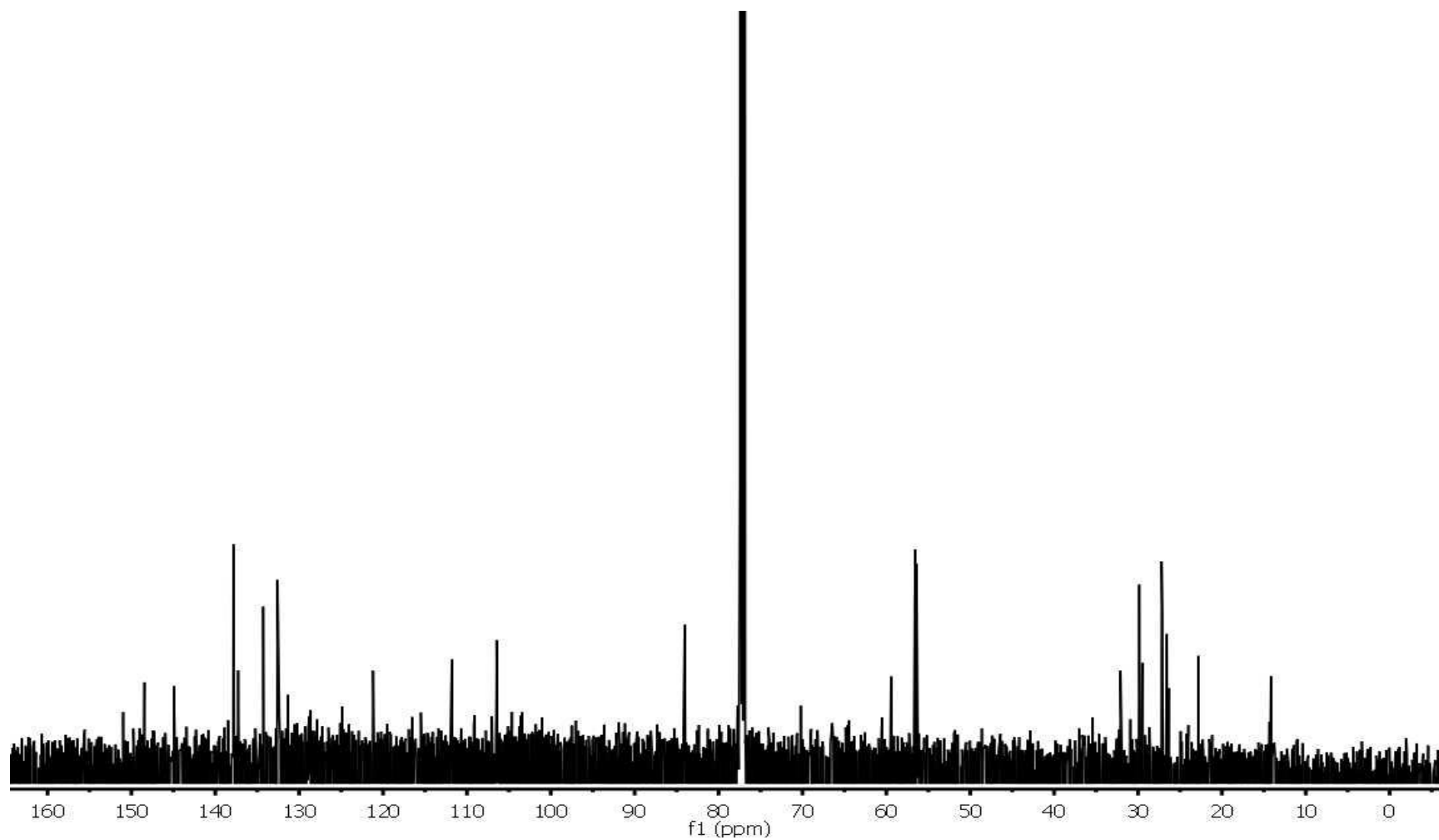

Supplement: Supplementary file 1 [file molecules-18-02438-s001.pdf]
